# Interface flexibility controls the nucleation and growth of supramolecular networks

**DOI:** 10.1038/s41557-025-01741-y

**Published:** 2025-02-13

**Authors:** Vincenzo Caroprese, Cem Tekin, Veronika Cencen, Majid Mosayebi, Navid Asmari, Tanniemola B. Liverpool, Derek N. Woolfson, Georg E. Fantner, Maartje M. C. Bastings

**Affiliations:** 1 Programmable Biomaterials Laboratory, Institute of Materials, School of Engineering, Ecole Polytechnique Fédérale Lausanne, Lausanne, Switzerland; 2 Laboratory for Bio- and Nano-Instrumentation, Interfaculty Bioengineering Institute, School of Engineering, Ecole Polytechnique Fédérale Lausanne, Lausanne, Switzerland; 3 School of Mathematics, University of Bristol, Bristol, UK; 4 Bristol BioDesign Institute, School of Chemistry, University of Bristol, Bristol, UK; 5 The Isaac Newton Institute for Mathematical Sciences, Cambridge, UK; 6 School of Biochemistry, University of Bristol, Bristol, UK

**Keywords:** Self-assembly, Molecular self-assembly, Two-dimensional materials, Organizing materials with DNA

## Abstract

Supramolecular networks are abundantly present in nature and, like crystalline materials, often develop from an initial nucleation site, followed by growth based on directional interactions between components. Traditionally, the binding strength and directionality of interactions is thought to dictate nucleation and crystal growth, whereas structural flexibility favours defects. Usually, macromonomers present multiple binding sites with relative intramolecular flexibility, but the effects of such flexibility on regulating network formation have been given little attention. Here we introduce the concept of ‘interface flexibility’ and demonstrate its critical importance in the nucleation and growth of supramolecular networks. As a model system, we use trisymmetric DNA-based macromonomers, which organize into hexagonal networks through weak π–π interactions at their tips. The directional nature and low spatial tolerance of π–π interactions mean that small shifts in orientation have a large effect on effective valency. We show that too much interface flexibility disrupts network formation, regardless of affinity. Tuning the interface flexibility greatly expands the available design space for synthetic supramolecular materials.

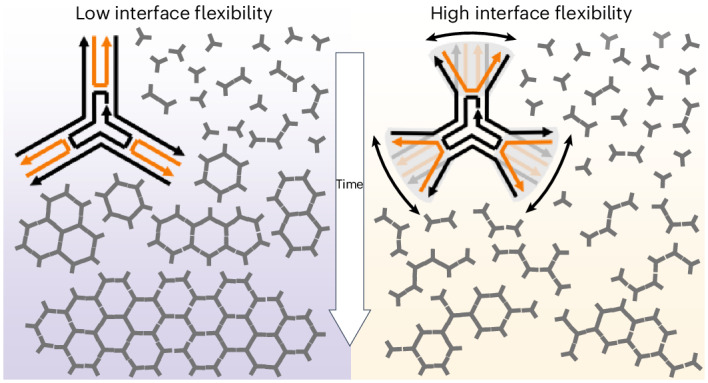

## Abstract (web summary)

Supramolecular ordered networks are formed through directional interactions of uniform macromonomer building blocks. Now it has been shown that, rather than intermolecular affinity, the flexibility of the binding interface (‘interface flexibility’) dominates the mechanism of self-assembly. This study provides an intuitive understanding of the role of interface flexibility in supramolecular self-assembly.

## Main

Dynamic yet controlled self-organization of macromolecules into temporal geometric domains allows for the engineering of functional interfaces for catalysis, materials and nanotherapeutics^1,2^. While challenging for engineers, such supramolecular networks are fundamental for many biological processes. For instance, the assembly of clathrin triskelions into polygonal patches enables cellular uptake^3–5^ and the organization of TRIM5a in hexagonal patterns on the HIV-1 capsid combats viral infection^6^. Traditionally, the main parameters used to design directional multivalent interactions are the binding strength (affinity) and the number of binding events (valency)^7^. Interestingly, both clathrin and TRIM5a use additional structural changes to transition from monomers into their characteristic geometric patterns^3,6^. Consequently, the nucleation of these networks is influenced by local changes in mechanical properties of the monomers. These mechanical properties influence the spatial tolerance of the intermolecular binding interface and thereby impact the systems’ capacity to acquire long-range order. While the structure–function connection of these biological phenomena is evident, their complex multicomponent environment limits a systematic manipulation of molecular parameters to explore the role of interface mechanics in nucleation and growth of supramolecular ordered networks.

Crystal formation is a complex process that involves both physical and chemical mechanisms, yet always starts with several molecules coming together to create a stable nucleation event^8^. This process is driven by many properties of the system, balancing concentration, diffusion and surface energies^9,10^. In traditional covalent crystals, the bonding between atoms is typically strong and directional, yielding a highly ordered and stable crystal structure^11^. Supramolecular crystals often show a more flexible and dynamic structure, attributed to the weaker noncovalent interactions and a larger macromolecular monomer unit cell out of which they are built^12,13^. This implies that structural mechanics of this macromolecular unit cell plays a critical role in the formation of supramolecular crystals.

Similar to nucleation in covalent crystals, noncovalent interactions in supramolecular crystalline and highly ordered materials have a directional component at the intermolecular interface^14^. A deepened understanding of the flexibility limits of bond directionality at the supramolecular interface could translate into novel engineering strategies to tailor the nucleation of such materials. To enable a detailed molecular analysis of the structural design parameters that influence the nucleation and growth mechanisms of supramolecular networks, modular monomers without complexity of biology are essential. While protein design and engineering approaches towards geometric networks have shown beautiful laboratory-made examples^15–18^, modulation of flexibility and affinity is not straightforward without substantially altering the monomer building block, and may come with unforeseen consequences^19–21^. Furthermore, directional interactions at protein–protein interfaces may engage a large number of amino acids and render the detailed understanding of local effects challenging. At micron length scales, colloidal supramolecular crystals guided by molecular recognition at the interface present impressive degrees of programmable design^22–25^, yet the effect of nuanced changes in structural mechanics get lost in the overall system dimensions.

Compared with protein-based monomers and colloids, DNA nanotechnology^26^ offers a predictable and modular platform to decouple the interplay between macromolecular flexibility and monomer–monomer affinity. When using synthetic DNA as material tool, structural flexibility can be controlled by the balance of single-strand versus double-strand sections, as well as the overall length compared with the persistence length of the DNA double helix^27^. Affinity can be manipulated through the length of single-strand complementary ends (‘sticky ends’) or the nucleotide sequence at the end of a ‘blunt-end’ double helix, as GC presents a stronger π–π stacking interaction than TA^28^. Utilizing the multivalency concept, a higher affinity interaction can be designed by systematic multimerization of adjacent DNA blunt ends. Combined, these characteristics make DNA an ideal model material to explore to what extend rigidity at the intermolecular interface guides the nucleation-growth mechanisms in supramolecular two-dimensional networks.

## Results and discussion

### Subtle structural changes yield varied network architectures

**Fig. 1:**
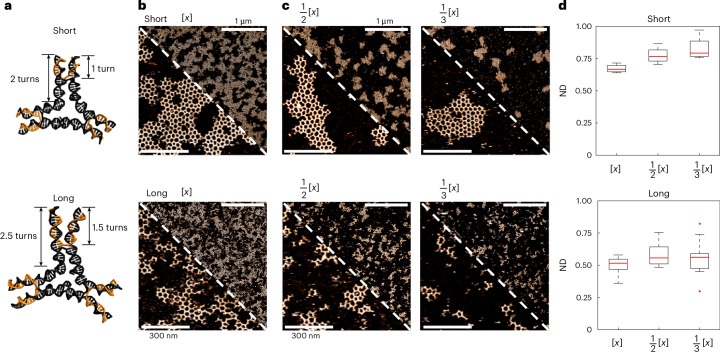
Steady-state assembly of lattices formed by short and long 3PS*.* **a**, DNA blueprint of the short and long monomers obtained from molecular dynamics simulations, indicating the overall and terminal arm lengths in double-helical turns. **b**, AFM images of the steady-state networks formed by the short (top) or long (bottom) monomers. **c**, AFM images of the steady-state networks formed by the short (top) or long (bottom) monomers at decreasing concentrations [*x*]. **d**, Weighted mean of the ND as a function of monomer concentration, using images from 12 regions across the mica surface, each with an area of 750 nm × 750 nm, for each condition tested. The box represents the interquartile range (IQR), with the lower and upper edges corresponding to the 25th and 75th percentiles. Whiskers extend to the smallest and largest data points within 1.5 times the IQR from the lower and upper quartiles, respectively. Outliers, defined as data points beyond this range, are shown as individual points. Upper scale bars, 1 µm; zoomed-in lower scale bars, 300 nm. Conditions: overnight incubation on mica at room temperature in the presence of 10 mM MgAc_2_. [*x*] is 6 nM for short and 3.8 nM for long 3PS (Supplementary Fig. 4). AFM *z*-colour bars are adjusted for optimal data presentation (lowest intensity corresponds to background at 0 nm, and highest intensity corresponds to DNA at approximately 2 nm).

DNA-based macromonomers assembled from a limited number of oligonucleotides (‘tiles’) have been designed and used to engineer a range of simple to highly complex supramolecular systems^29^. We used the established DNA three-point-star (3PS) motif as starting point, which allowed us to build upon existing work on the supramolecular crystalline self-organization of DNA tiles^30^ (Supplementary Fig. 1). This macromolecule presents a threefold rotational symmetry and consists of seven ssDNA strands—three unique sequences—organized in three four-arm junctions (Fig. 1a). The conventional approach to obtain supramolecular crystals from DNA tiles is via the display of short ssDNA terminal ends that enable ‘sticky-end’ hybridization with their sequence complement. These networks assemble in solution and show long-range order with very few defects^31^. However, the use of sticky ends comes at the cost of kinetic control and renders the analysis of self-assembly mechanisms related to monomer design obsolete. In a blunt-ended variant, the interaction interface consists of a multivalent array of π–π stacking units, presented by the nucleobases. When multiple DNA helices are present in parallel, the distance from a DNA cross-over junction affects the spatial freedom at the end of a DNA helix extension^32^. The geometric alignment of multiple of these weak noncovalent interactions allows for a sufficiently strong directional force to self-assemble into two-dimensional networks. However, they are also reversible and this inherent dynamics permits targeting the nucleation-growth mechanism of network formation^33^.

To explore the impact of monomer mechanics on the growth and lattice structure, we made two 3PS variants: one with arms of two DNA turns (Fig. 1a, ‘short’) and a second 3PS with added 0.5 turn extensions per arm (Fig. 1a, ‘long’). The modification generates a larger star motif in terms of absolute size and corresponding global flexibility, but also the spatial tolerance of the arm termini (terminal flexibility) is increased. This terminal flexibility results from the distance after the Holliday junction in the middle of the 3PS arm (Fig. 1a), allowing the two parallel double helices to have more freedom and move independently. We let both systems assemble overnight in aqueous buffer solution on mica (Supplementary Fig. 2) and imaged the steady-state networks by atomic force microscopy (AFM). Clear differences between the steady-state assemblies of the short and long 3PS were observed (Fig. 1b). The short monomers predominantly formed large hexagonal networks, whereas the long monomers assembled into smaller, elongated structures. To explore whether the difference between the designs was related to the monomer concentration, we imaged both systems over a range of concentrations (Fig. 1c and Supplementary Fig. 3). Measurements at higher concentrations led to surface crowding and multilayer assembly (Supplementary Fig. 4). For all dilutions, we consistently observed large islands for short 3PS and many small structures for long 3PS.

We developed a tailored detection algorithm in MATLAB^34^ to reliably identify each 3PS monomer centre, assembled island and the polygons present in a frame (Methods and Supplementary Fig. 5). By considering each island as a network of polygons, we applied the concept of network density (ND)^35^ to quantify the degree of ideal radial growth within the assembled islands (Supplementary Fig. 6). ND values close to 1 indicate observed islands that are close to an ideal crystalline network. As ND drops, the monomer organization is increasingly less crystalline, but, for instance, show more elongated assemblies. Application of the ND to all steady-state results confirmed the presence of two distinct supramolecular network architectures (Fig. 1d). The short 3PS follow the assembly of a radial crystalline network much closer than the long variant and ND approaches 1 at the lowest concentration. We deduce from this that radial growth is hindered by coalescence of multiple islands at the higher concentrations. By contrast, the low ND value characteristic of the elongated assemblies seen for the long 3PS is concentration independent. Thus, the small change in arm geometry and associated terminal flexibility has a consequential impact on the global network self-assembly.

### Rigidity and affinity cooperate for crystalline organization

**Fig. 2:**
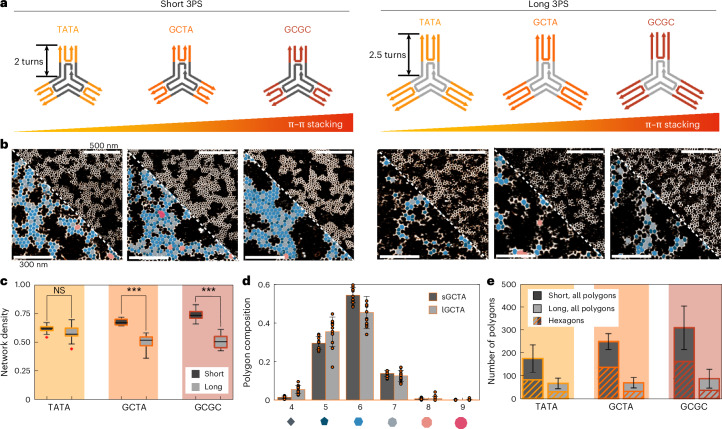
Quantifying the effect of intermolecular affinity on network crystallinity*.* **a**, A 3PS library, where each monomer has a unique combination of an arm length (2 or 2.5 turns) and a terminal base pair (TATA, GCTA or GCGC). The blunt-end π–π interaction strength increases from left to right (from TATA to GCGC). **b**, AFM images of the assembly in steady-state (after 24 h) for each 3PS design (top right triangle). Higher magnification and overlay of the polygon identification algorithm (pentagons, dark blue; hexagons, blue; heptagons, light grey) (bottom left triangle). AFM *z*-colour bars are adjusted for optimal data presentation (the lowest intensity corresponds to background at 0 nm and the highest intensity corresponds to DNA at approximately 2 nm). **c**, ND of all 3PS assemblies showcasing a moderate effect of affinity in short 3PS systems yet marginal impact in long 3PS systems. A two-sided *t*-test was applied to each short–long pair with the same terminal bases (***P < 0.001). *P* values for TATA, GCTA and GCGC pairs are 0.097, 7.9 × 10^−9^ and 7.7 × 10^−10^, respectively. The box represents the IQR, with the lower and upper edges corresponding to the 25th and 75th percentiles. Whiskers extend to the smallest and largest data points within 1.5 times the IQR from the lower and upper quartiles, respectively. Outliers, defined as data points beyond this range, are shown as individual points. **d**, Polygon distributions for short (sGCTA) versus long (lGCTA) assemblies. **e**, Average count of all polygons, with the hexagon contribution highlighted by the striped surface. Each data point in **c**–**e** represents a 750 nm × 750 nm region, with a total of 12 data points derived from two separately prepared mica surfaces presented in each case. Error bars represent mean values ± s.d.

Next, we considered whether changes in intermolecular binding affinities could overcome the negative effects on lattice propagation caused by structural flexibility. For DNA-based macromonomers, these affinities can be tuned by the number of parallel double helices (two in each 3PS arm) or the sequence of terminal nucleotides participating in the blunt-end π–π stacking interactions^28^. When fully surrounded by neighbours in a hexagonal arrangement, 3 × 2 directional π–π interactions are formed, stabilizing otherwise weak binding through the strength-in-numbers principle of multivalency. Since the π–π stacking energy is defined by the chemical structure of the DNA nucleobase, the overall binding affinity between monomers can be controlled through the sequence of the terminal nucleotides. Therefore, we modified the original long and short 3PS motifs, which had GCTA as terminal nucleotide pairs, to present either TATA or GCGC (Fig. 2a and Supplementary Figs. 7 and 8). These modifications directly affect the π–π stacking energy, expected to range from Δ*G* ~2 kcal mol^−1^ for TATA, to Δ*G* ~6.8 kcal mol^−1^ for GCGC^28^. We wondered whether, besides affecting global network formation, the choice of terminal nucleotide identity would also impact the local polygonal composition.

The six 3PS variants were assembled and steady-state AFM showed consistent patterns within the groups of short and long monomers (Fig. 2b and Supplementary Fig. 9), suggesting that changes in intermonomer affinity had little or no impact on the overall network structure. From the ND values, increasing the intermonomer affinity for short 3PSs slightly enhanced the radial crystal organization, whereas no clear effect was apparent for the long 3PSs (Fig. 2c). Interestingly, the weakest short 3PS (TATA) approaches the organization of the long 3PSs, yet the strongest long 3PS (GCGC) does not approach that of the short 3PS system. We hypothesized that the high surface density was biasing network coalescence. To better understand the behaviour of the weakest short 3PS system (TATA), we analysed its steady-state assembly at lower concentrations (Supplementary Fig. 10). The observed lattices behaved differently to GCTA, forming a greater number of islands while retaining a radial pattern. This behaviour may cause premature coalescence, potentially resulting in the lower ND values at the concentrations shown in Fig. 2c. Taken together, while the terminal nucleotide couple changes the interaction affinity, the mechanical properties of the monomer dominate the formation dynamics.

For a quantitative analysis of the internal polygon composition, we extended our image-processing algorithm to automate the detection and labelling of polygonal geometries from three to nine monomers in short GCTA versus long GCTA assemblies (Fig. 2d and analyses of TATA and GCGC in Supplementary Fig. 11). This revealed that hexagonal organization is prevalent, with pentagonal and heptagonal assemblies responsible for the majority of ‘defects’. Similar heterogeneity in polygon distribution has been observed in clathrin networks and synthetic peptide architectures, and can be explained by a moderate structural flexibility within the monomers^3,4,19^. Plotting the hexagonal content within all polygons leads to two conclusions (Fig. 2e): first, the total amount of assembled polygons is notably higher for the short compared with the long 3PS molecules, indicating a favoured network growth mechanism. Second, the hexagonal content within the short 3PS is moderately dependent on the terminal affinity, with short GCGC and short GCTA forming the most crystalline network, as judged by the fewest polygonal defects and highest ND (Fig. 2e). This suggests that rigidity and affinity cooperate to create a more-crystalline organization.

### Real-time imaging of network formation reveals two mechanisms

**Fig. 3:**
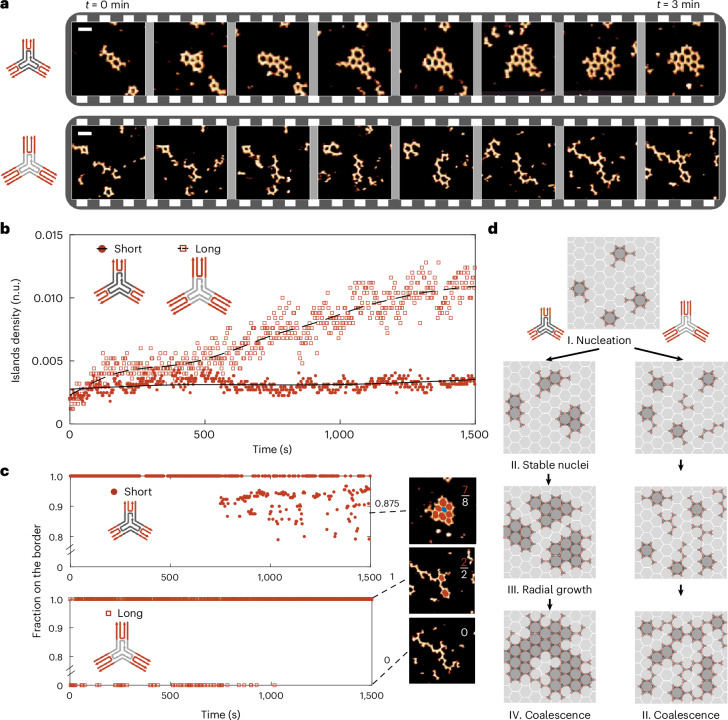
HS-AFM detection of two distinct growth mechanisms*.* **a**, Time-lapse of short (top) and long (bottom) 3PS self-assembly over 3 min, highlighting the difference in nucleation. Snapshots are taken from full-length time series. Scale bars on the top left, 50 nm. AFM *z*-colour bars are adjusted for optimal data presentation (the lowest intensity corresponds to background at 0 nm and the highest intensity corresponds to DNA at approximately 2 nm). **b**, Detected island density over time (25 min imaging) using tailored particle tracking algorithms for long and short 3PS. The black lines are fitted smoothing splines. n.u., normalized units. **c**, Quantification of radial crystal island growth through detection of fraction of polygons on a border (coloured red) over the total number of polygons. When no polygons are present, the ratio is taken to be 0. **d**, A schematic representation of the nucleation-growth self-assembly mechanism of short 3PS and coalescence-based elongated island formation of long 3PS.

The steady-state data for the 3PS variants suggest fundamental differences in the assembly mechanism between the short and long monomers. To visualize and analyse the mechanisms of assembly from the earliest interactions, we used high-speed AFM (HS-AFM)^36–38^. By using photothermal off-resonance tapping (PORT) mode^38,39^, the effect of tip interference with the self-assembly process was reduced^39^. With a frame rate of 0.4 fps, the organization of 3PS macromolecules into the typical hexagonal patterns was observed from the moment of monomer injection (Fig. 3a and Supplementary Videos 1–6), focusing on the initial phases of nucleation.

Upon qualitative inspection, we directly noticed the short and long 3PS monomers had different self-assembly pathways (Fig. 3a). The short 3PSs formed a small number of large islands indicative of stable initial nucleation events followed by radial growth. By contrast, multiple, small and elongated islands observed in the long 3PS samples suggest a lack of stable nucleation. Both behaviours were observed independent of the 3PS terminus (for example, affinity). To quantify these differences, we identified all islands and particles present over time using the aforementioned image analysis (Fig. 3b and Supplementary Fig. 12, with additional examples in Supplementary Figs. 13–21). The density of islands remained constant for the short 3PS, whereas for long 3PS the number of islands grew, all while the density of monomers on the surface steadily increases due to the continuous surface adsorption of monomers from the bulk (Supplementary Fig. 12). This confirms that incoming monomers attach to a stable nucleus and contribute to the growth of the supramolecular crystal for the short 3PS, whereas incoming long 3PS monomers do not nucleate and eventually crowd the surface and coalesce into larger elongated structures.

We next counted the number of polygons present on the border of the biggest island and expressed it as a ratio in respect of the total number of polygons in that island (Fig. 3c). When the network grows, an increasing number of polygons will be in the centre of an island (Fig. 3c, blue), causing a decrease of this border ratio. Following an initial nucleation time, which for the short 3PS systems was ≈10 min, the first centre-monomers (for example, border ratio <1) started to appear. Subsequently, the border fraction further decreased, which is a strong confirmation of radial island growth from an initial stable nucleus. The long 3PS showed a very different profile as during the time of the experiment, all monomers remained present at a border, either as polygon (‘1’) or in an elongated polymeric conformation (‘0’).

Combining HS-AFM and the particle-detection analyses, we propose the following kinetic pathways for the assembly of the two 3PS systems (Fig. 3d). Considering the short 3PS system first. As π–π stacking is a weak interaction, the blunt-ended 3PS motifs only interact laterally when on a surface where they can diffuse freely as monomers. Due to the parallel alignment of DNA double helices in the arms of the 3PS motifs, a multivalent array of π–π interactions is presented. Together with the structural tri-symmetry, stability of the assembly increases by multimerization of the π–π stacking interactions leading to the formation of polygonal nuclei. These nuclei are less diffusive than the 3PS monomers because the nuclei have larger contact areas and more interactions with the mica surface. This reduced mobility allows the formation of stable nuclei and subsequent growth of supramolecular crystals. By contrast, for the long 3PS, visibly no stable nuclei are formed, which prevents the transition to a radial growth phase. Eventually, however, as the density of monomers at the surface increases, the many small nuclei coalesce to create elongated island structures (Fig. 3d), as observed in steady-state analysis. Lastly, we note that both systems show continuous making and breaking of polygons. The noncovalent intermolecular bonds together with the structure of the monomer allows for perpetual self-reorganization. This is particularly clear in the short 3PS systems, where larger networks are present and reshuffling between polygon types can be observed, though the overall polygon fractions do not change. Unfortunately, while high speed, the sampling rate of HS-AFM is not sufficient to study these self-healing kinetics.

### Interface flexibility determines stable nucleation

**Fig. 4:**
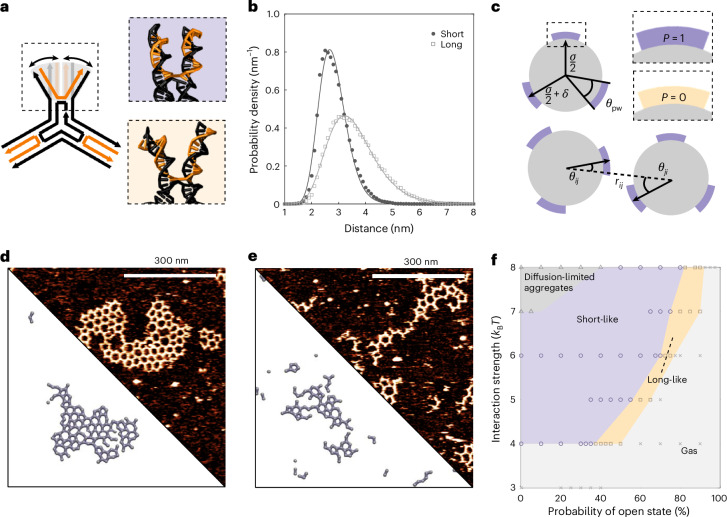
Demonstration of interface flexibility*.* **a**, A representation of interface flexibility related to the mobility of the peripheral 3PS segments following the Holiday junction. The upper box represents an aligned, ‘closed’ configuration that allows directional binding and the lower box represents a wider, ‘open’ configuration that is unavailable for binding. **b**, The probability distribution of the end-to-end arm distances at the π–π interface obtained from the oxDNA molecular dynamics simulations of the 3PS monomers. The purple area highlights the region where the arms are in a closed state and yellow is used for the open state. **c**, A patchy-particle model used for Monte Carlo simulations, highlighting the patch state with variable *p* (*p* = 1 for closed state and *p* = 0 for open state), and the *P*_open_ the probability that a patch has a state *p* = 0. Other model parameters defined as in the classical Kern–Frenkel model, where *σ* is the diameter of the hard-core repulsion, *δ* is the attraction range and *θ*_pw_ is the patch width. *r*_*ij*_ is the center-to-center distance between the two interacting particles, *i* and *j*. See Supplementary Information for details. **d**,**e**, Comparison of simulation results, bottom and experimental measurements, top of short 3PS (**d**) and long 3PS (**e**). For AFM images, the lowest intensity corresponds to background at 0 nm and the highest intensity corresponds to DNA at approximately 2 nm. The selected frames are taken from simulation trajectories with similar average ND values as in the experiments. **f**, An approximate state diagram as a function of the probability of open state (that is, when *p* = 0) and the interaction strength. The dark grey region shows the diffusion-limited aggregation regime, purple/orange shows the short-like/long-like regime and the light grey shows the gas (mostly single particles) phase. Each data point represents a set of conditions simulated, with length dimensions normalized to our short design. The state diagram was obtained for *σ* = 1, *δ* = 0.038 and *θ*_pw_ = 0.44.

The observed difference in stable nucleus formation suggested the interface between the two types of monomer to be critically different. These interfaces are formed by a pair of double-stranded DNA helices that make up each arm of the monomers. Considering the strong directional nature of the π–π stacking, the strength of interaction between two particles is directly affected by the possible orientations that two termini can assume: it will be maximised when precisely parallel and aligned and quickly fall off as the termini are oriented in different directions, to a breaking point where just one weak π–π bond can form. The latter situation effectively renders this arm of the 3PS ‘defective’ for binding, for example, the 3-valency turns into a 2-valency (Supplementary Fig. 22). This lets us define two possible extreme states for an arm: ‘open’ if the double helices are point in different directions and ‘closed’ if sufficiently parallel to maximize π–π interactions (Fig. 4a). To assess the possible distributions of short and long 3PS in these states, we used oxDNA^40,41^ (Supplementary Figs. 23 and 24) and a series of molecular dynamics^42^ simulations (Supplementary Methods). The sampled probability distribution functions of the end-to-end distance between DNA helices in an arm (Fig. 4b) show that the short 3PS presents a narrower distribution (~2 nm, centred around ~2.7 nm) compared with the long 3PS (~6 nm, centred at ~3.2 nm). As the spatial tolerance to form two simultaneous π–π stacking interactions is 1.5–2.5 nm, the arms of short 3PS are almost four times more likely to be oriented in a bond-forming conformation than the arms of long 3PS. Therefore, the chance that long 3PS makes productive interactions leading to stable nuclei is greatly reduced compared with the short 3PS units. This change in interface flexibility provides a potential underlying explanation for the different mechanisms of supramolecular assembly observed for long versus short 3PS.

We turned to Monte Carlo simulations^42^ to explore the effect of interface flexibility and spatial tolerance of the π–π interface, and their impact on nucleation in greater detail. A patchy-particle model was used to describe the 3PS motif as a particle made up of a repulsive core and three attractive sites equally distributed on its perimeter (Fig. 4c). This is a common strategy used for the study of directional interactions in macromolecular systems^43–46^. The aforementioned ‘closed’ and ‘open’ states were introduced into a classical Kern–Frenkel^47^ potential through the concept of the ‘state’ of a patch, where the state variable assumes a value of 1 when representing closed arms (indicating that the interface is available for binding) or 0 when the interface is open (detailed information about the model in Supplementary Methods). Similar to an Ising model^48,49^, each interface can switch states through a dedicated move following preassigned probabilities. Through this approach, we decouple the free energy of interaction into two components: an energetic contribution from patch–patch interactions and an entropic contribution from interface flexibility.

We explored the parameter space representing the ensemble of interface flexibility by varying the state-switching probabilities and the strength of the interaction. This allowed us to recover states that are qualitatively and quantitatively similar to those obtained in our steady-state AFM measurements (Fig. 4d,e). Using the ND and island size as observables to distinguish between different assembly mechanisms, we constructed a complete assembly diagram (Fig. 4f). Besides single molecules or small aggregates states (similar to a two-dimensional ‘gas’)^50^, we observe states that match our long 3PS (anisotropic elongated clusters) and short 3PS (compact isotropic clusters) labelled long-like and short-like, respectively. Regardless of the affinity, we always find an interval of flexibility where nucleation-growth is impaired. Excessive flexibility renders a 3PS functionally defective, preventing it from interacting with all three arms simultaneously. For linear assemblies to be formed, a 3PS needs to behave like a 2 + 1PS or 1 + 2PS (the second number is indicative of the ‘defective’ arm). Each observed assembly snapshot can thus be reproduced using a carefully designed mixture of effective 1/2/3 valent 3PS macromonomers (Supplementary Fig. 22). However, we experimentally only have a one-component system, with all three interfaces identical. This confirms that interface flexibility alone defines the mechanism of supramolecular nucleation-growth mechanism. To the best of our knowledge, this is a unique case, since elongated structures are typically observed only when approaching the diffusion-limited aggregation conditions (that is, in the limit of infinite interaction strength)^51–53^.

### Restoring the interface rigidity returns stable nucleation

**Fig. 5:**
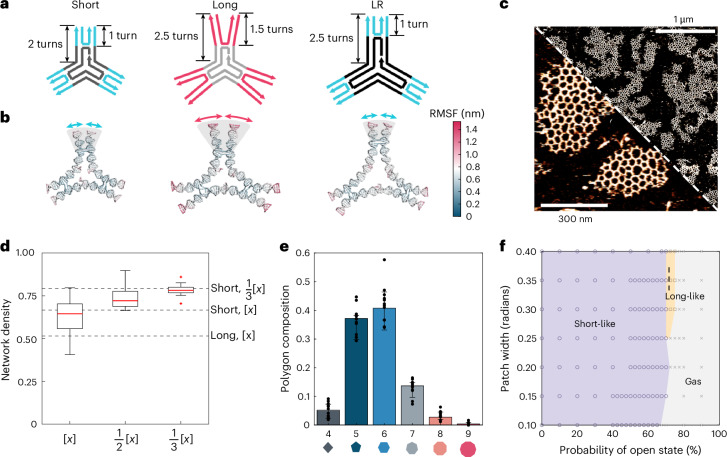
Restoration of interface rigidity*.* **a**, Design of LR 3PS and comparison of local and global flexibility with the original short 3PS and long 3PS designs. **b**, Representative frames from oxDNA simulations of the 3PS, coloured by the average root mean square fluctuation (RMSF). **c**, Steady-state AFM of LR 3PS, showing a restored radial large network assembly. AFM *z*-colour bars are adjusted for optimal data presentation (the lowest intensity corresponds to background at 0 nm and the highest intensity corresponds to DNA at approximately 2 nm). **d**, ND of LR 3PS assemblies at decreasing concentrations ([*x*] = 3.8 nM); dashed lines represent references for short rigid (SR) and long flexible (LF) monomers. The box represents the IQR, with the lower and upper edges corresponding to the 25th and 75th percentiles. Whiskers extend to the smallest and largest data points within 1.5 times the IQR from the lower and upper quartiles, respectively. Outliers, defined as data points beyond this range, are shown as individual points. The AFM images of 1/2× and 1/3× are shown in Supplementary Fig. 28. Additional AFM images for all concentrations are shown in Supplementary Fig. 29. **e**, Polygon distributions for LR 3PS assemblies, using images from 12 regions across the mica surface, each with an area of 750 nm × 750 nm, for each condition tested. The error bars represent mean values ± s.d. **f**, An approximate phase diagram as a function of probability to be in the open state and the patch width for *σ* = 1 and *δ* = 0.038. Purple, short-like phase; orange, long-like phase; grey, gas phase. Each data point represents a set of conditions simulated.

For the initial long 3PS design, the arms were terminally extended, keeping the central DNA strand, and with it the cross-over point in the arms, identical to the short design (Figs. 1a and 2a). As such, two additional sites of flexibility are present in the long 3PS design: a global arm flexibility due to the extended overall length and the prolonged distance from the cross-over point results in a considerable local flexibility^32^ at the 3PS periphery (Fig. 5a,b). To reduce the interface flexibility and attempt to reintroduce a nucleation-growth profile for long 3PS, we moved the position of the cross-over point. This gave a new ‘long-rigid’ (LR) 3PS monomer (Fig. 5a,b and Supplementary Fig. 25). Moving the DNA cross-over position in this way does not alter the overall monomer geometry, but it should change structural rigidity, exemplary of the strategic modularity present within DNA nanotechnology^32^. OxDNA simulations of the three 3PS designs (short, long and LR) showed changes in global and peripheral flexibility, and confirmed that th*e* LR 3PS monomer displayed a restored interface rigidity similar to short 3PS (Fig. 5b and Supplementary Fig. 26). Since the overall arm length is the same as the long 3PS, the global flexibility corresponds to that found in the original long 3PS.

We repeated all assembly experiments with the LR 3PS and radially grown large networks were visible (Fig. 5c), with a ND score matching that of the short 3PS (Fig. 5d). Contrarily, polygonal quantification confirmed the distribution to be more similar to that observed for the original long 3PS (Fig. 5e); however, with increased contributions of pentagons and higher-order polygons. Thus, while the peripheral rigidity restores the interface flexibility and mechanism of nucleation and growth, the crystallinity of these networks is still dominated by global flexibility.

Finally, we investigated whether the restoration of the radial growth could have resulted from a confounding factor such as the increased global flexibility, caused by the increased arm length. This increases the effective width of the interface that, in our patchy-particle model, translates to an increased value of the patch width parameter. We explored the parameter space associated to the patch width and the probability of being in an open state, representing the interface flexibility (Fig. 5e). All simulations used an interaction strength of 6 *k*_B_*T* (Supplementary Methods) that was previously shown to cover the ensemble of possible states (Fig. 4f). The phase diagram shows two regimes: above 0.25 rad both network morphologies are present, with boundaries almost parallel to the axis of the patch width. Contrarily, for more globally rigid systems, a direct transition from short-like to gas phase for *P*_o_ of between 60% and 77.5% was seen. This implies that above a certain global flexibility threshold, the long-like state is always observable and no other form of dependency can be inferred. Our systems are represented by a patch width of ~0.3 rad (based on the polygon fractions in Supplementary Fig. 27), therefore the differences observed in our experiments are controlled by interface flexibility rather than global flexibility.

## Conclusions

Our study shows that a subtle change in local structural flexibility can have a dramatic effect on a systems’ global self-assembly, guided by interface flexibility. The highly directional nature of π–π stacking interactions results in a rapid fall off with distance. This low spatial tolerance means that slight shifts in orientation can strongly affect both the directional assembly and the effective valency of particles utilizing these interactions. The observed on/off switching of self-assembly due to fine changes in local density of binding units suggests a direct parallel to super-selectivity^54^, here present in the most minimal form of multivalency, for example, divalent interactions. Using Monte Carlo simulations, we demonstrated the critical effect of interface flexibility as the defining parameter of network formation. In our analysis, we decomposed the interaction free energies into energetic and entropic components, enabling an intuitive presentation of the system. This partitioning simplifies the interpretation of results, emphasizing that interface flexibility can dominate over energetic attraction for supramolecular network formation. While demonstrated with DNA-based monomers in this study, these fundamental insights translate beyond the DNA helix and promise to provide an invaluable aid to the future design of more complex building blocks. The extent to which interface flexibility will moderate self-assembly depends on the nature of each macromolecular building block. Evidently, fully rigid macromonomers will not be affected and systems with a very high valency of interacting binding units will experience an overriding effect of overall avidity.

Flexible-to-rigid transitions in biological macromonomers are surprisingly common, resulting from lateral interactions between monomers as seen in the example of the clathrin triskelion as well as via the addition of helper proteins in the case of the TRIM5a organization. While constructed from very different biological buildings blocks, these systems only assemble into geometric networks when the macromonomers are temporarily rigidified. Indeed, clathrin’s ability to ‘shapeshift’ is thought to contribute to the diversity of lattice structures and therefore its multiple cellular functions^55^. With insights obtained in this study, we may start to look at biological transitions of flexibility and order with new appreciation for structural protein monomer design and their transient interfaces. Reversibly, the intentional introduction of interface flexibility can be used strategically when the formation of large supramolecular networks is not desired or should be disrupted (for example, in the case of plaques, fibres, amyloids or even viral (dis)assembly). Exploiting the global and local rigidity/affinity balance at (biological) interfaces presents new opportunities for the engineering of dynamic cellular nanotherapies and controlled growth—or disruption—of molecular networks.

## Methods

Full materials and methods and additional characterization can be found in Supplementary Information.

### Preparation of DNA 3PS motifs

For each 3PS, ssDNAs were mixed at the assigned molar ratios in the annealing buffer containing 5 mM TRIS (Bio-Rad), 1 mM EDTA (ITW Reagents) and 10 mM MgAc_2_ (abcr GmbH) to give a final solution of 0.6 µM DNA motif in 50 µl (pH adjusted to 8.0). The DNA solutions were annealed at 80 °C for 5 min, 60 °C for 10 min, cooled from 60 °C to 20 °C by 1 °C every 10 min, 20 °C for 10 min and stored at 4 °C.

### Static AFM imaging

First, 40 µl of DNA tile at the designated concentration was deposited on a freshly cleaved mica (grade V1, Ted Pella). Mica was placed in a Petri dish containing 1 ml of water to create a humid environment and sealed with Parafilm. The sample was left overnight for self-organization on the mica surface before imaging. All AFM images were acquired in tapping mode in liquid on a Cypher VRS (Asylum Research Inc.) using a BioLever mini cantilever (BL-AC40TS-C2, Olympus).

### HS-AFM imaging

Images were acquired on a home‐built small cantilever AFM with photothermal excitation as described elsewhere^38,39^. All images were acquired in PORT mode at frequencies of 100 kHz using AC10DS cantilevers (Olympus). First, 50 µl of 10 mM MgAc_2_ was injected close to the cantilever in the dedicated channel of the cantilever holder. The cantilever was then approached to the surface and the surface was scanned to check for contaminations. The cantilever was retracted from the surface, the buffer extracted from the holder and 50 µl of the DNA 3PS solution (~3.8 nM for long and ~6 nM for short) was injected and imaged immediately. The sample was scanned at a line rate of 100 Hz (256 lines × 256 pixels) unless otherwise specified. The set point was kept at the lowest level required for proper tracking.

### Automated analyses of assembled networks

Extraction of information from AFM images has been carried through a custom MATLAB^34^ routine of (1) opening and standard AFM artefacts removal, (2) segmentation and skeletonization, (3) polygon and particle detection, and (4) collection of observables per connected component (island). For the statistics related to island properties, a threshold of 24 particles has been applied to take an island in consideration.

### OxDNA simulations

We prepared a vHelix^56^ in Maya^57^ model for all compounds with GC TA as the terminal base couple, which was subsequently converted it in oxDNA-compatible^40,41^ formats for coordinates and topology using TacoxDNA^58^. Unless stated otherwise, simulations follow standard settings for the base dependent forcefield (oxDNA2) and a salt concentration of 1 M. The pipeline involved a minimization of 1 × 10^6^ steps using the steepest descent algorithm. Eight sets of molecular dynamics were run in NVT ensemble, where we progressively increased the temperature using the following ramp (in °C): 1, 1, 10, 10, 20, 20, 25 and 25. Until this moment, we kept the restraints between base pairs using the standard settings generated with the related tool in OxView^59,60^ and used a time step of 5 × 10^−4^. We then set the time step to 1 × 10^−3^ and proceeded with a last simulation at 25 °C for 5 × 10^6^ steps, where we halved the strength associated to the restraints before starting our production run at the same temperature without base pair restraints. During the production runs, configurations were stored every 5 × 10^5^ steps. The resulting files were converted to psf, pdb and mdcrd files using an in-house Python script. Analysis, snapshots and measures were then performed using VMD^61^ (http://www.ks.uiuc.edu/Research/vmd/mailing_list/vmd-l/att-32062/system_freeze.vmd) and MATLAB.

### Patchy-particle simulations

Monte Carlo simulations were run on a modified version of the engine developed by Hedges (http://vmmc.xyz/), available online (https://github.com/mosayebi/PatchyDisc). A modified version of the Kern–Frenkel potential^47^ was implemented to take into account patch states and adhesion to mica. A detailed formulation of the potential and specific parameters used in each set of simulations are given in Supplementary Information. The recorded trajectories were rendered as images using an in-house Python script and VMD. The analysis of the frames was then performed in MATLAB following similar procedures as for the experimental AFM data (without step 1). Labelling of the states to construct the phase diagrams shown in Figs. 4 and 5 was done based on the last 20 frames recorded in each condition. The assignment of the states was performed by evaluating ND as the principal metric and island size as a secondary determinant. Details about the potential used, the specifics of the simulations and the construction of the phase diagrams are reported in Supplementary Notes.

## Online content

Any methods, additional references, Nature Portfolio reporting summaries, source data, extended data, supplementary information, acknowledgements, peer review information; details of author contributions and competing interests; and statements of data and code availability are available at 10.1038/s41557-025-01741-y.

## Supplementary information

Supplementary Information Supplementary Methods, Notes, Figs. 1–29 and Table 1.

Supplementary Video 1 Dynamic self-assembly of short GCGC monitored by HS-AFM.

Supplementary Video 2 Dynamic self-assembly of long GCGC monitored by HS-AFM.

Supplementary Video 3 Dynamic self-assembly of short GCTA monitored by HS-AFM.

Supplementary Video 4 Dynamic self-assembly of long GCTA monitored by HS-AFM.

Supplementary Video 5 Dynamic self-assembly of short TATA monitored by HS-AFM.

Supplementary Video 6 Dynamic self-assembly of long TATA monitored by HS-AFM.

Supplementary Video 7 Patchy-particle simulation corresponding to short monomer. The specific simulation parameters used are: *P*_o_ = 0.8, *ε* = 8, *θ*_pw_ = 0.44, *σ* = 1 and *δ* = 0.038.

Supplementary Video 8 Patchy-particle simulation corresponding to long monomer. The specific simulation parameters used are: *P*_o_ = 0.9, *ε* = 8, *θ*_pw_ = 0.44, *σ* = 1 and *δ* = 0.038.

## Data Availability

All data that support the conclusions in this paper are present in the main text or the supplementary materials. Source data for this paper are available via Zenodo at 10.5281/zenodo.11545939 (ref. ^62^).

## Code availability

The simulation engine is available online via GitHub at https://github.com/mosayebi/PatchyDisc. The routine used for image analysis of both AFM and simulation trajectories is provided via GitHub at https://github.com/VinceCar/Branched_particles_images_analyzer.
