# Supplementary material for: Interface flexibility controls the nucleation and growth of supramolecular networks
Source: Nat Chem. 2025 Feb 13;17(3):325–33. doi: 10.1038/s41557-025-01741-y (PMC11882452; doi:10.1038/s41557-025-01741-y)
Supplement: Supplementary file 1 — Supplementary Methods, Notes, Figs. 1–29 and Table 1. [file 41557_2025_1741_MOESM1_ESM.pdf]

nature chemistry

Supplementary information https://doi.org/10.1038/s41557-025-01741-y

Interface flexibility controls the nucleation and growth of supramolecular networks

In the format provided by the authors and unedited

Table of Contents

Extended Methods 3

Oligonucleotides 3

Purification of DNA tiles 3

Native PAGE. 3

Further details on analyses of AFM images 4

Detailed protocol for patchy-particle simulations. 5

Detailed protocol for phase diagram construction. 7

Specifications for simulations of isolated monomers in solutions. 8

Specifications for simulations of isolated monomers on surface 8

Supplementary Figures and Tables 10

Supplementary Fig. 1. Native PAGE (6%) analysis of the formation of (a) short GCTA and (b) long GCTA DNA 3PS 10

Supplementary Fig. 2. The evolution of the sGCTA and lGCTA 3PS assembly. after (a,d) 1 day, (b,e) 2 days and (c,f) 4/9 days. 12

Supplementary Fig. 3. AFM images of short 3PS (a-c) and long 3PS (d-f) at [x] (a, d), 1/2[x] (b, e), and 1/3[x] (c, f) concentrations. 13

Supplementary Fig. 4. DNA concentration for AFM experiments. 14

Supplementary Fig. 5. Image processing and analysis flowchart 15

Supplementary Fig. 6. Ideal island for the calculation of Network Density (ND) 16

Supplementary Fig. 7. Native PAGE (6%) analysis of the formation of (a) short TATA and (b) long TATA 3PS. 17

Supplementary Fig. 8. Native PAGE (6%) analysis of the formation of (a) short GCGC and (b) long GCGC 3PS. 17

Supplementary Fig. 9. AFM images of sTATA (a), sGCGC (b), lTATA (c), and lGCGC (d). 18

Supplementary Fig. 10. sTATA at half concentration. 19

Supplementary Fig. 11. Polygon compositions at the steady state for (a) TATA and (b) GCGC. 19

Supplementary Fig. 12. Dynamic self-assembly of short and long GCGC. 20

Supplementary Fig. 13. Dynamic self-assembly of lGCGC 3PS. Example 2. 21

Supplementary Fig. 14. Dynamic self-assembly of sGCTA 3PS. Example 1. 21

Supplementary Fig. 15. Dynamic self-assembly of sGCTA 3PS. Example 2. 22

Supplementary Fig. 16. Dynamic self-assembly of sGCTA 3PS. Example 3. 22

Supplementary Fig. 17. Dynamic self-assembly of lGCTA 3PS. Example 1. 23

Supplementary Fig. 18. Dynamic self-assembly of lGCTA 3PS. Example 2. 23

Supplementary Fig. 19. Dynamic self-assembly of sTATA 3PS 24

Supplementary Fig. 20. Dynamic self-assembly of lTATA 3PS. Example 1. 24

Supplementary Fig. 21. Dynamic self-assembly of lTATA 3PS. Example 2. 25

Supplementary Fig. 22. Monte Carlo simulation snapshots of patchy-particles with a 3-valent:2-valent ratio of 1:2 (a), 1:3 (b), and 0:1 (c)..25

Supplementary Fig. 23. OxDNA simulation frames of (a) short, (b) long and (c) long rigid DNA 3PS in solution. 26

Supplementary Fig. 24. OxDNA simulation frames of (a) short, (b) long and (c) long rigid DNA 3PS confined on the surface..27

Supplementary Fig. 25. Native PAGE (6%) analysis of the formation of long rigid (LR) 3PS. 28

Supplementary Fig. 26. Probability distribution function of 3PS end-to-end distances based on OxDNA simulations...29

Supplementary Fig. 27. Polygon composition in the patchy particle simulations. 29

Supplementary Fig. 28. AFM images of the long rigid 3PS self-assembly at lower concentrations. 30

Supplementary Fig. 29. AFM images of long rigid at [x] (a), 1/2[x] (b), and 1/3[x] concentrations. 30

Supplementary Table 1. DNA motifs and the base sequences of the strands. 31

Extended Methods

Oligonucleotides.

All DNA tiles designed in this study are based on the original 3-point-star (3PS) motif[1]. The modifications on the DNA sequences of the 3PS motif were made following the principles presented by Seeman[2]: All 4 nucleotide-long subsequences of individual DNA strands are (1) unique, (2) not self-complementary (e.g. TGCA) and (3) includes both purine and pyrimidine. The same rule applies for the 4 nt-long subsequences that are on a continuous DNA duplex but span a junction. All oligonucleotides used in this study were either bought from Integrated DNA Technologies, Inc. or synthesized in-house on OligoMaker (TAG Copenhagen) following standard synthesis protocols. The sequences are listed in Supplementary Table 1.

Purification of DNA tiles.

Preparation methods of individual DNA tiles can be found in the main text Materials and Methods. The annealed product was loaded on a 3% agarose (Sigma) gel and the gel was run at 60 V for 150 minutes in an ice-cooled water bath. The running buffer contained 0.5x TBE (Thermo Scientific, 44.5 mM Tris, 44.5 mM boric acid and 1mM EDTA; pH 8.0) and 10 mM $MgAc_2$. The band corresponding to the DNA motif was excised with a surgical blade and loaded in a Freeze 'N Squeeze gel extraction spin column (Bio-Rad). The column was centrifuged for 20 minutes at 3000 x g and 4 ^{o}C. To replace the buffer with the storing buffer (same with the annealing buffer), the flow through was collected and pipetted to a Vivaspin 500, MWCO 3000 (Sartorius). After an initial spin at 3000 x g for 30 minutes, 300 μL of 1x storing buffer was added to the solution and centrifuged again at 3000 x g for 90 minutes. This last step was repeated one more time to ensure that the buffer is replaced.

Native PAGE.

6% polyacrylamide gels were prepared following standard protocols. The gels were run at 120 V for 50 min in 0.5xTBE. The gels were stained with SYBR Gold (Sigma) and imaged with ChemiDoc MP (Bio-Rad).

Further details on analyses of AFM images.

After opening of an image through a third-party developed script[3] in MATLAB[4], the routine includes standard practices, such as: median line differences removal, adaptive thresholding to identify background, polynomial surface fitting of the background and median line removal using background as reference[5]. We iterated the process a second time after resizing the image to a standard pixel width of 0.34 nm. Finally, we applied a gaussian filter with standard deviation of 2 pixels and capped the final values between 2% and 98% of the values in the image.

For segmentation and skeletonization, a first enhancement of the contrast between foreground and background is obtained by applying a custom filter based on a kernel of radii equal to the size of the particle of interest and intensity that would reduce progressively from the center to the border, in a way that would take into account the different orientations that a particle can assume. Initial thresholding and average filtering of the background are then applied to further enhance the image. Afterwards, a preliminary skeletonization step based on the watershed transform is performed. Using the preliminary skeleton, we perform a second, more precise thresholding to obtain a mask. This mask is then further processed to enhance holes, remove full areas and fill scars. For the steady state images, when automatic filling of scars would fail due their excessive thickness, we would resort to manual correction of the original image and restart the process.

Finally, the mask is refined through morphological operators and a 4-connected skeleton is extracted through a standard skeletonization algorithm.

The polygon and particle detection part are based on the hypothesis that segments in an image connect the centers of two particles. Therefore, we proceed with the identification of segments in the skeletonized image considering that a segment is either: (1) shared by two polygons, (2) isolated or (3) forming an angle with another one. For each isolated connected component in the foreground, we try to divide it in segments based on principle 1 and then apply a Ramer-Douglas-Peucker algorithm (based on the native one implemented in MATLAB) in order to identify all the vertices in each segmented line. To facilitate the vertex detection algorithm, when possible, we define a list of initial candidate vertices, consisting of branched points and the point that is furthest away from the center of mass. Each vertex is the center of a 3PS-particle and polygons can be

identified by counting their vertices. Finally, we check how many polygons and particles (and eventual properties like being in contact with the background) a connected component has and stores it in dedicated matrices.

Prior to these steps, for the videos analysed in Supplementary Fig. 16 and Supplementary Fig. 19, a rescaling along the acquisition axis of the images was performed in order to compensate for a noticible hysteresis. Areas were recalcualted accordingly.

Detailed protocol for patchy-particle simulations.

The current version of the engine used is made available online at https://github.com/mosayebi/PatchyDisc. Main modifications from the engine developed by Hedges[6] include: a dedicated routine to parse input conditions for each simulation from a json file, the definition of a new potential and variables, and implementations of moves to change the states of the patch.

The pairwise interaction between two particles is given by (see Fig. 4c)

$$U_{(r_{ij},\theta_i,\theta_j,p_i,p_j)} = \begin{cases} \infty, & r_{ij} < \sigma \\ -\varepsilon p_i p_j, & \sigma < r_{ij} < \sigma + \delta \wedge \theta_i < \theta_{pw} \wedge \theta_j < \theta_{pw} \\ 0, & otherwise \end{cases} \quad \text{Supplementary Eq. 1}$$

where, r_{ij} is the center-to-center distance between the two interacting particles, i and j; σ is the diameter of a particle; δ is the radial width for the interaction; the term ε represent the patch-patch binding energy; θ_i is the smallest angle formed by the direction of any of the patches in particle i and the line connecting the centers, similarly θ_j is the smallest angle formed by the direction of any of the patches in particle j and line connecting the centers; θ_{pw} represents the maximum angle at which the interaction can happen; p_i and p_j are respectively the states of the aforementioned patches in i and j and they can either assume a value of 0 or a value of 1. This formulation corresponds to a standard Kern-Frenkel (KF) potential[7], with the extra condition that both patches only interact when they are in closed state.

In addition to the modified KF potential, we consider a surface penalty term (W) that opposes changes occuring to a particle when at least one of its patches is involved in a patch-patch bond. This term is motivated by our experimental observation that interacting monomers engage with the mica surface more effectively than isolated monomers. As a result, isolated monomers exhibit high mobility and cannot be resolved, whereas dimers are clearly observed in AFM images. The surface penalty term mimics the effect of energy barriers between potential wells on a lattice, which limit the diffusion of adsorbed atoms[8,9]. The energy change associated with the particle-particle energy and the penalty to disengage the surface to pass from a configuration "*a*" to "*b*", $\Delta H_{a \to b}$, can then be expressed as:

$$\Delta H_{a \to b} = \Delta U_{a \to b} + W[U_a < 0] \qquad \text{Supplementary Eq. 2}$$

The moves in our Monte Carlo (MC) simulation are either translation/rotation of a particle or changing the state of a single patch (which is attempted 3 times more often than the translation/rotation move). To ensure detailed balance, we accept moves according to the Metropolis rule, calculated as:

$$\Delta G_{a \to b} = \Delta H_{a \to b} + \Delta P_{a \to b}, \qquad \text{Supplementary Eq. 3}$$

where $\Delta P_{a \to b}$ is the free energy change resulting from switching the patch state while moving from *a* to *b*, based on the probability of the patch being in "open" state, P_o, when not interacting with others (in our case, for a particle *i* in a simulations of *N* partilces, $\sum_{0,j \neq i}^{N} U_{ij} = 0$).

Since we have only 2 possible states the specific formulation of the second term is

$$\Delta P_{a \to b} = \begin{cases} 0, & p_a = p_b \\ \log\left(\frac{P_o}{1-P_o}\right), & p_a = 0 \wedge p_b = 1 \\ \log\left(\frac{1-P_o}{P_o}\right), & p_a = 1 \wedge p_b = 0 \end{cases} \qquad \text{Supplementary Eq. 4}$$

Where p_a represent the state of the patch in state *a* and p_b represent the state of the patch in state *b*.

The units of length in the simulations are normalized using as reference length the diameter of a particle (16 nm in our case), the energy is normalized by $k_B T$, where k_B is Boltzmann's constant, T is the thermodynamic temperature and angles are measured in radians.

Simulations are performed in 2D placing 1500 particles over an area of 150x150 normalized units (~1.6x1.6 μm^2), matching densities measured for our low-density images. The total number of sweeps is at least 2.4e8 for each simulation. The interaction width is set at 0.038, implying that a π-π stacking would have a width of ~0.6 nm[10]. Respecting the conditions of the one bond per patch regime[10], θ_{pw} has been tested between 0.1 and 0.40 radians (used for Fig. 5). ε has been tested between 2 to 8 (used for Fig 4). In Fig 5, ε was kept at 6 $k_B T$. W, when a particle is interacting with another particle, has a value of 6 $k_B T$, otherwise 0. Particle positions and orientations are initialized randomly, while each patch state is initialized to 0.

Detailed protocol for phase diagram construction.

To visualize the simulation results, we constructed two pseudo phase-diagrams that depict the probability of an arm being in an open state (P_o) against either interaction strength (ε, Figure 4f) or patch width (θ_{pw}, Figure 5e). The same methodology was applied to build these diagrams, regardless of whether the secondary variable was interaction strength or patch width.

Initially, for each simulated value of the secondary variables, we fitted the Network Density (ND) values as a function of P_o using a sigmoid curve. Points where ND was zero were excluded during the fitting process. Additionally, points at P_o values lower than the P_o corresponding to the maximum ND were excluded if they had fewer than 10 islands of at least 24 monomers in any of the frames used for the statistics. These instances were classified as "diffusion-limited" and were not included in the sigmoid fitting process. This classification was confirmed through visual inspection of the trajectories.

The final trend line was constructed using the fitted sigmoid curve for ND values above zero and applying a flat line at y=0 for ND values of zero.

Each point on the trend line was then classified as follows:

- "Diffusion-limited": If P_o was less than that of the first data point retained for the fitting.
- "Short-like": Points where the ND is at 90% of the sigmoid plateau.
- "Long-like": Points that are not "short-like" but still have P_o below the last observed data point with ND greater than zero.
- "Gas-like": All other cases.

Finally, the pseudo phase-diagrams were constructed by organizing these classified points in the plane defined by P_o and the secondary variables.

Specifications for simulations of isolated monomers in solutions.

The protocol followed the one reported in Materials and Methods. Production runs consisted of 1.85e8 steps. 9 replicas were performed for each monomer, respectively: short, long and long rigid. During analysis we discard the first 1e7 steps and considered frames collected every 5e5 steps. In Supplementary Fig. 23 are reported for each compound characteristic configurations and the RMSF computed over all the trajectories.

Specifications for simulations of isolated monomers on surface.

In a similar fashion to what has been done in Materials and Methods, we prepared flat *de novo* designs of our monomers. However, these models have been used to select the starting configurations from the trajectories simulated in the previous section as follows: for each monomer, we selected as starting configuration the one that would have the lower RMSD compared to the manually constructed flat models. Afterwards, we manually aligned the starting structures to a plane perpendicular to the y axis using OxView[11,12], and then applied the rest of the protocol described in Materials and Methods. In this context, we run simulations while implementing two repulsion planes, confining the compound in a layer of around 2 nm, starting with a force constant of 0.3 for the minimization. Additionally, we increased the steps in the minimization process to 7.5e6. Throughout the equilibration NVT simulations we slowly increased the force of the repulsion plane, by setting the associated parameter to: 0.3, 0.6, 0.9, 1.2, 1.5, 1.8 and 2.1. The last was then kept throughout the last equilibration run and production.

Production runs consisted in 1.35e8 steps. 12 replicas were performed for each monomer. During analysis we would discard the first 1e7 steps and considered frames collected every 5e5 steps.
In Supplementary Fig. 24 are reported for each compound characteristic configurations and the RMSF computed over all the trajectories; end to end distance are plotted in Supplementary Fig. 26.

References

1. He, Y., Chen, Y., Liu, H., Ribbe, A. E. & Mao, C. Self-assembly of hexagonal DNA two-dimensional (2D) arrays. *J Am Chem Soc* **127**, 12202–12203 (2005).
2. Seeman, N. C. Nucleic acid junctions and lattices. *J Theor Biol* **99**, 237–247 (1982).
3. Fricke, E. L. Gwyddion File Importer. https://www.mathworks.com/matlabcentral/fileexchange/32893-gwyddion-file-importer (2011).
4. Inc., T. M. MATLAB version: 9.13.0 (R2022b). Preprint at (2022).
5. Nečas, D. & Klapetek, P. Gwyddion: an open-source software for SPM data analysis. **10**, 181–188 (2012).
6. Hedges, L. O. LibVMMC.
7. Kern, N. & Frenkel, D. Fluid-fluid coexistence in colloidal systems with short-ranged strongly directional attraction. *Journal of Chemical Physics* **118**, 9882–9889 (2003).
8. Baibuz, E. *et al.* Migration barriers for surface diffusion on a rigid lattice: Challenges and solutions. *Comput Mater Sci* **146**, 287–302 (2018).
9. Skaug, M. J. *et al.* Single-molecule diffusion in a periodic potential at a solid–liquid interface. *Soft Matter* **10**, 753–759 (2014).
10. Rovigatti, L., Russo, J. & Romano, F. How to simulate patchy particles. *European Physical Journal E* **41**, (2018).
11. Poppleton, E. *et al.* Design, optimization and analysis of large DNA and RNA nanostructures through interactive visualization, editing and molecular simulation. *Nucleic Acids Res* **48**, E72–E72 (2020).
12. Bohlin, J. *et al.* Design and simulation of DNA, RNA and hybrid protein–nucleic acid nanostructures with oxView. *Nat Protoc* **17**, 1762–1788 (2022).

Supplementary Figures and Tables

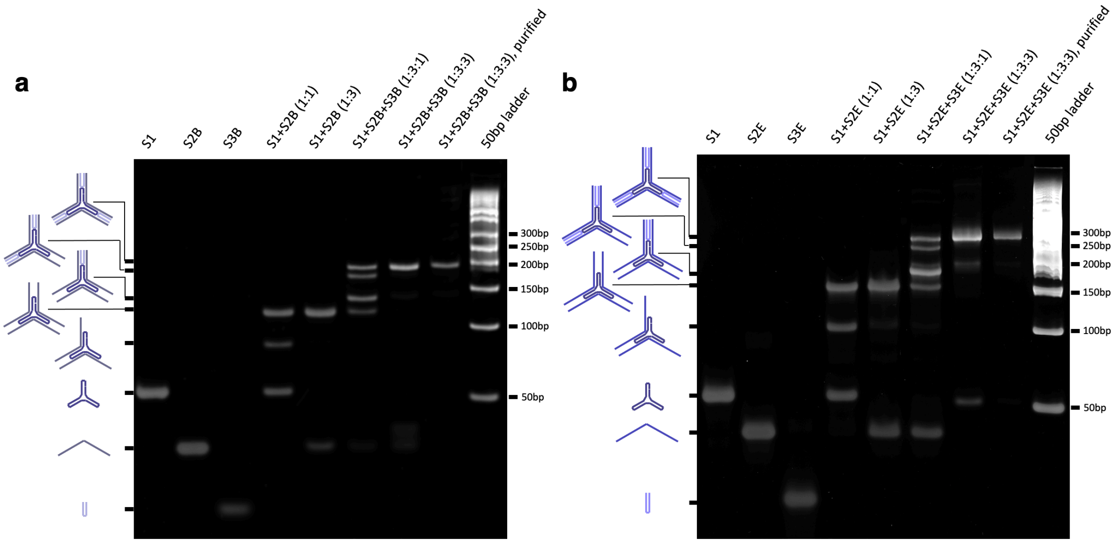

Supplementary Fig. 1. Native PAGE (6%) analysis of the formation of (a) short GCTA and (b) long GCTA DNA 3PS.

The compositions of the samples and the structures corresponding to each band are shown above and on left of the gels respectively.

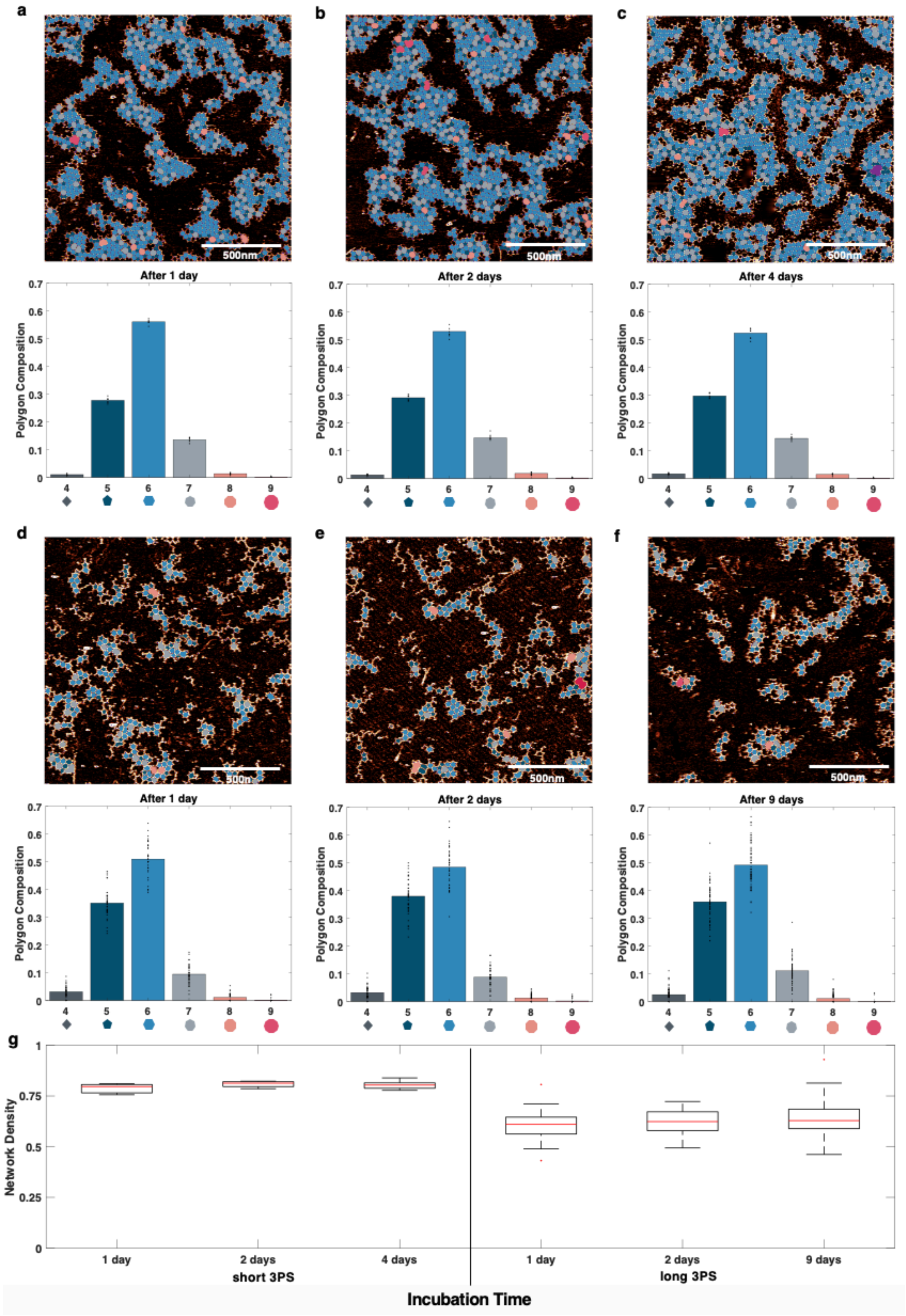
a
b
c
500nm
After 1 day
After 2 days
After 4 days
Polygon Composition
4
5
6
7
8
9
d
e
f
After 9 days
g
Network Density
1 day
2 days
4 days
9 days
short 3PS
long 3PS
Incubation Time

Supplementary Fig. 2. The evolution of the sGCTA and lGCTA 3PS assembly. after (a,d) 1 day, (b,e) 2 days and (c,f) 4/9 days.

a-c, The AFM images of the sGCTA assembly (on top) and the corresponding polygon distribution (on bottom) after 1 day (a), 2 days (b) and 4 days (c). **d-f,** The AFM images of the lGCTA assembly (on top) and the corresponding polygon distribution (on bottom) after 1 day (d), 2 days (e) and 9 days (f). **g,** Weighted average Network Density of sGCTA and lGCTA as a function of incubation time. The box represents the interquartile range (IQR), with the lower and upper edges corresponding to the 25th and 75th percentiles. Whiskers extend to the smallest and largest data points within 1.5 times the IQR from the lower and upper quartiles, respectively. Outliers, defined as datapoints beyond this range, are shown as individual points.

Bars present a weighted mean based on the number of monomers observed in an image. Error bars represent mean values +/- standard deviation. Each black dot represents the fraction of a polygon in a unique 750 nm x 750 nm region. The box plots in panel g include the data of the same 750 nm x 750 nm regions. After a day of incubation, the distribution of polygons remains constant, prompting us to opt for a one-day incubation period on mica.

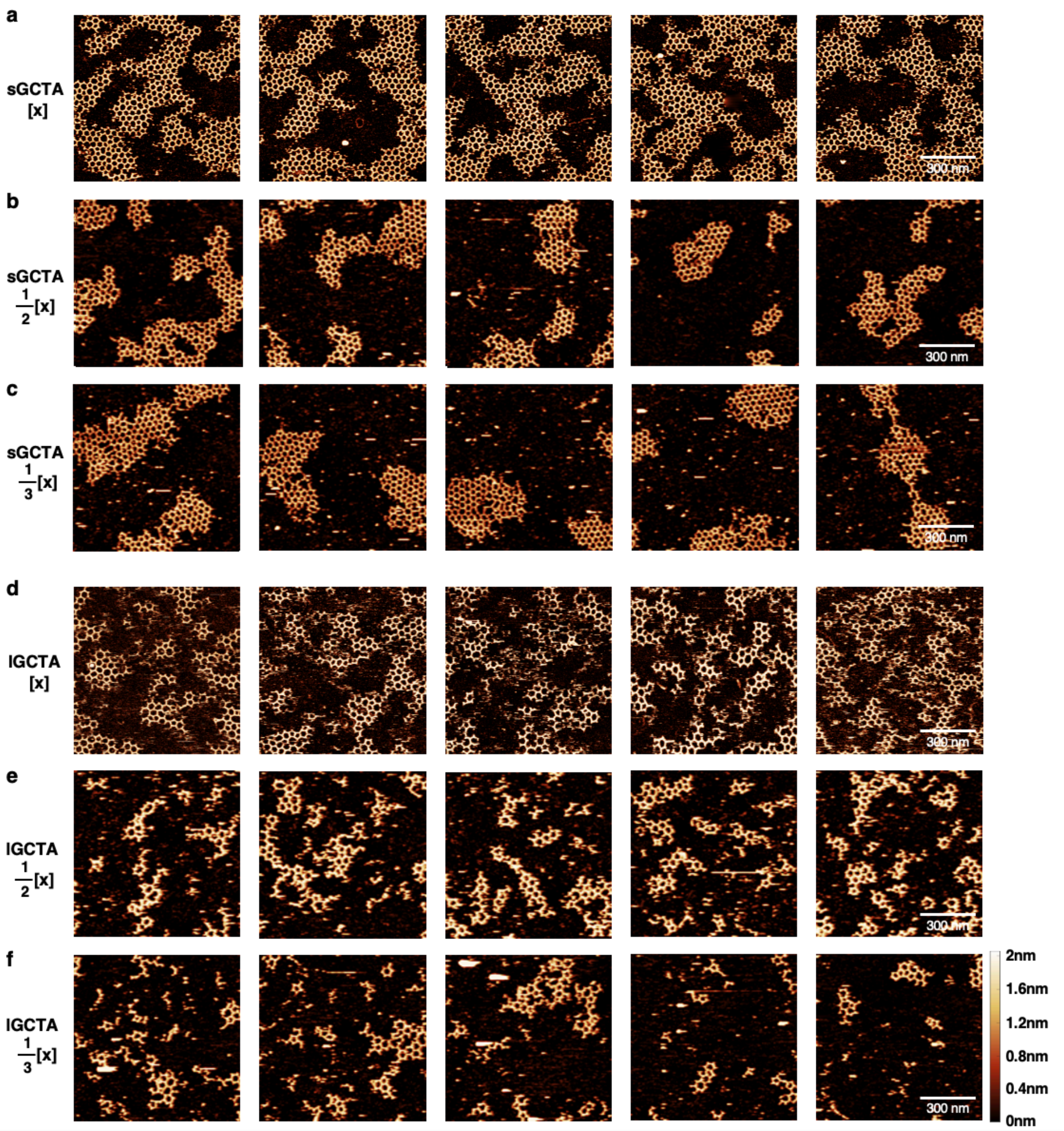

Supplementary Fig. 3. AFM images of short 3PS (a-c) and long 3PS (d-f) at [x] (a, d), 1/2[x] (b, e), and 1/3[x] (c, f) concentrations.

Examples of images used in the anaylsis given in Fig. 1. The colorbar for height representation provided in this figure is representative for all AFM images presented in this study. Each AFM image exclusively features the DNA 3PS motif, with a characteristic height of 2 nm. The contrast has been subtly adjusted for individual AFM images to optimize data presentation, following established practices in the literature. [x] is 6nM for sGCTA and 3.8nM for lGCTA.

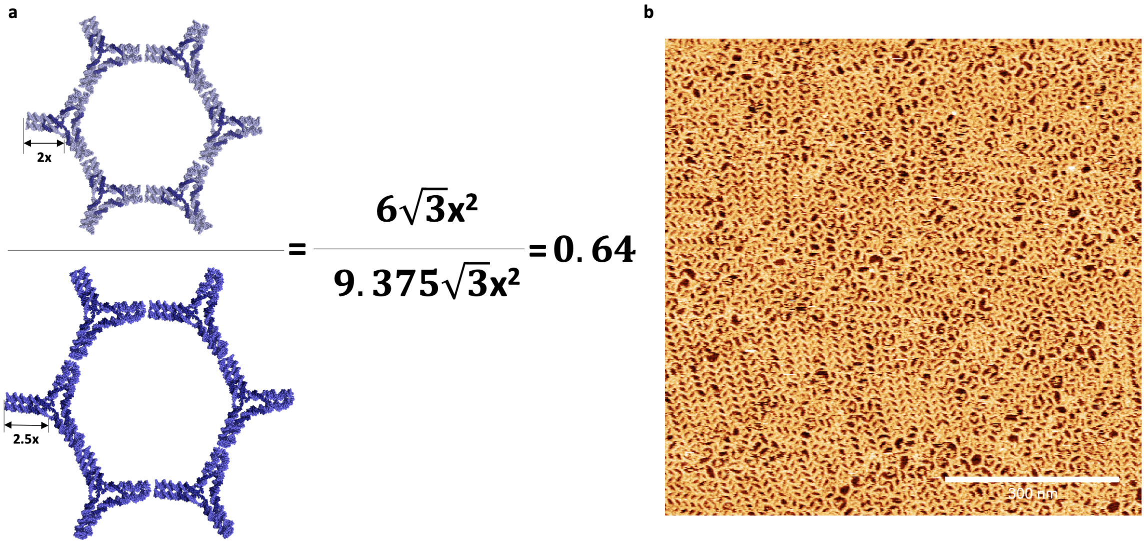

Supplementary Fig. 4. DNA concentration for AFM experiments.

a, Long 3PS concentration. **x** indicates a turn of dsDNA. To cover the surface as much as short 3PS, 0.64 x 6nM = 3.84nM of long 3PS was used in the experiments. **b,** Overcrowding. In the cases of excessively high concentration, some blunt-end interactions are disrupted and the 3PS pack in a denser final form that minimizes the void between them.

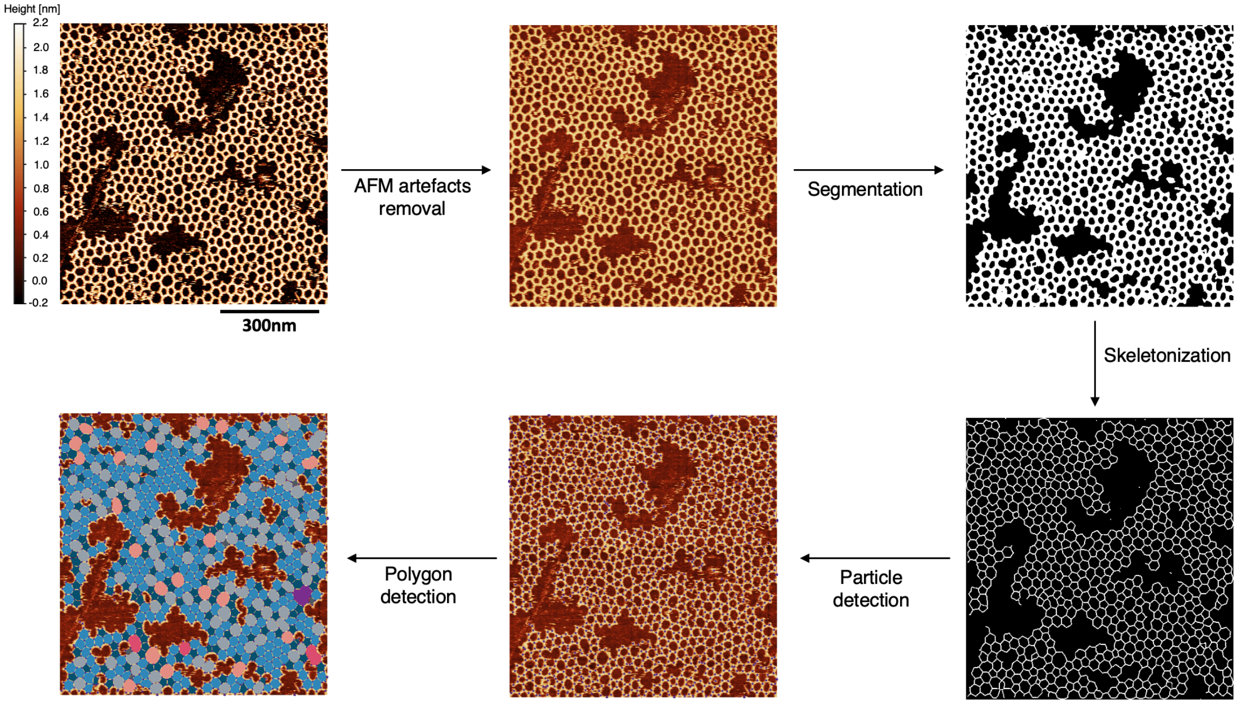

Supplementary Fig. 5. Image processing and analysis flowchart

The typical image processing and analysis workflow includes (1) AFM artefact removal, (2) segmentation, (3) skeletonization, (4) particle detection, and (5) polygon detection.

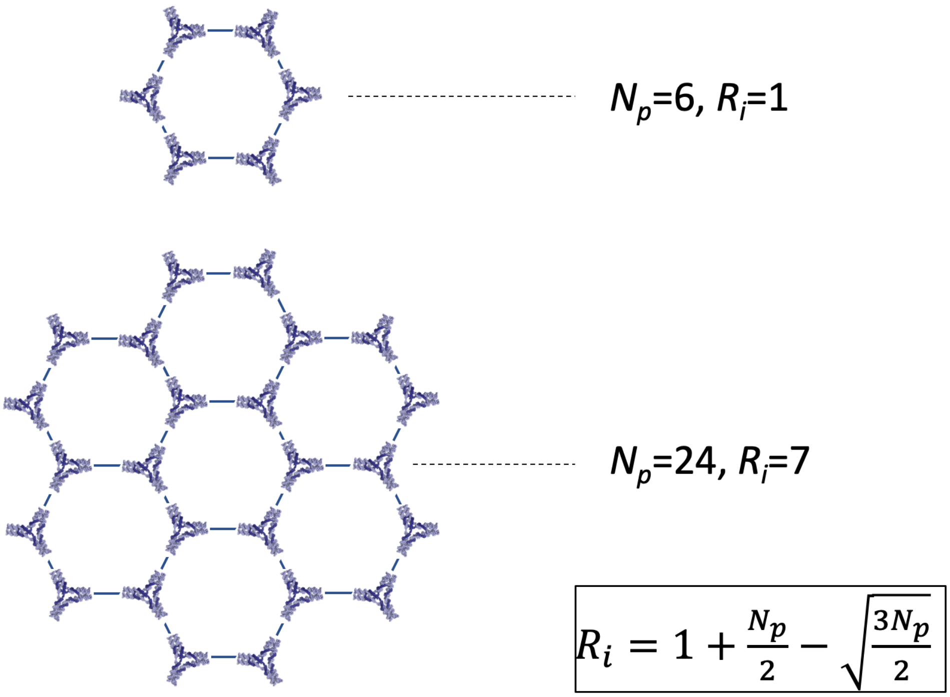

Supplementary Fig. 6. Ideal island for the calculation of Network Density (*ND*).

ND offers a quantifying measure of connectivity and organizational structure while intrinsically adjusting for the size of the network and its connectivity potential. Thereby, it provides a resilient measure against the finite size effects, matching the demands of our system. We model our islands as networks with vertices representing polygons and edges denoting adjacency. For our case, *ND* was expressed as the ratio between the number of observed polygons in an island and the number of polygons in an ideal island formed by the same number of monomers (N_p) arranged in a hexagonal symmetry and assembled by radial growth (R_i).

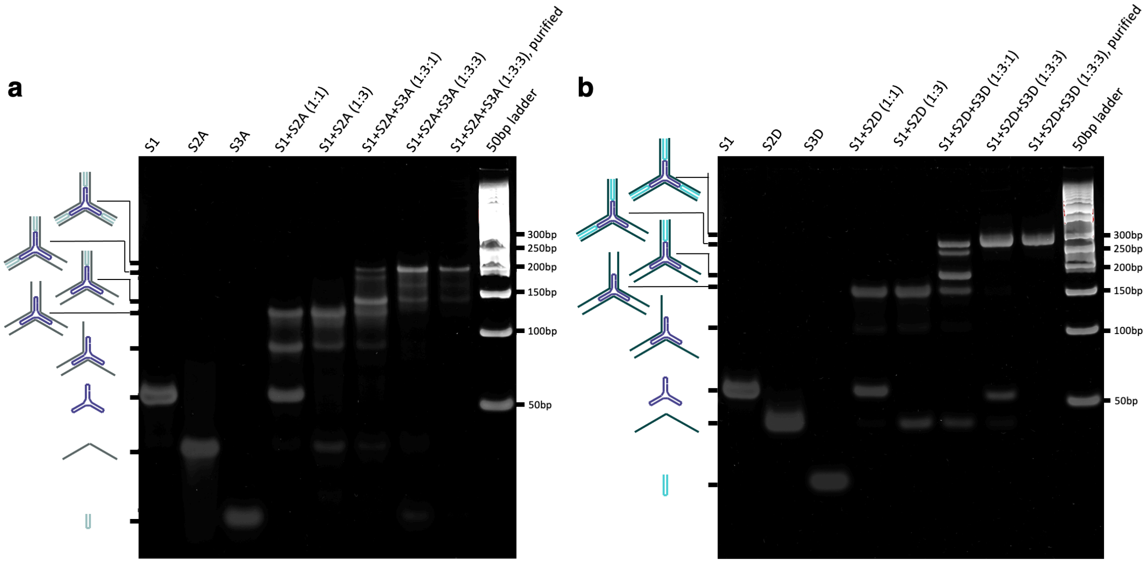

Supplementary Fig. 7. Native PAGE (6%) analysis of the formation of (a) short TATA and (b) long TATA 3PS.

The compositions of the samples and the structures corresponding to each band are shown above and on left of the gels respectively.

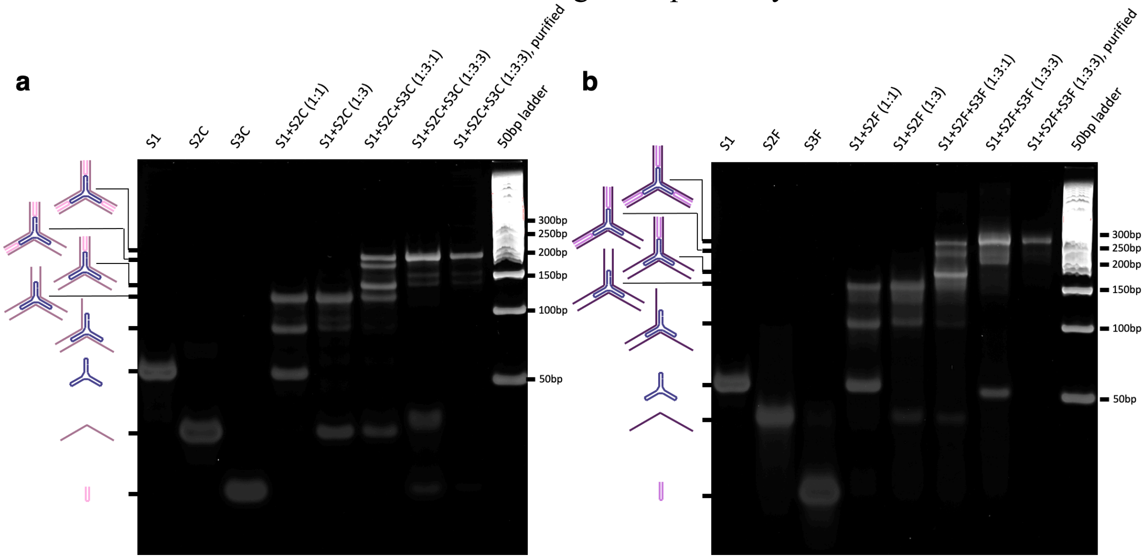

Supplementary Fig. 8. Native PAGE (6%) analysis of the formation of (a) short GCGC and (b) long GCGC 3PS.

The compositions of the samples and the structures corresponding to each band are shown above and on left of the gels respectively.

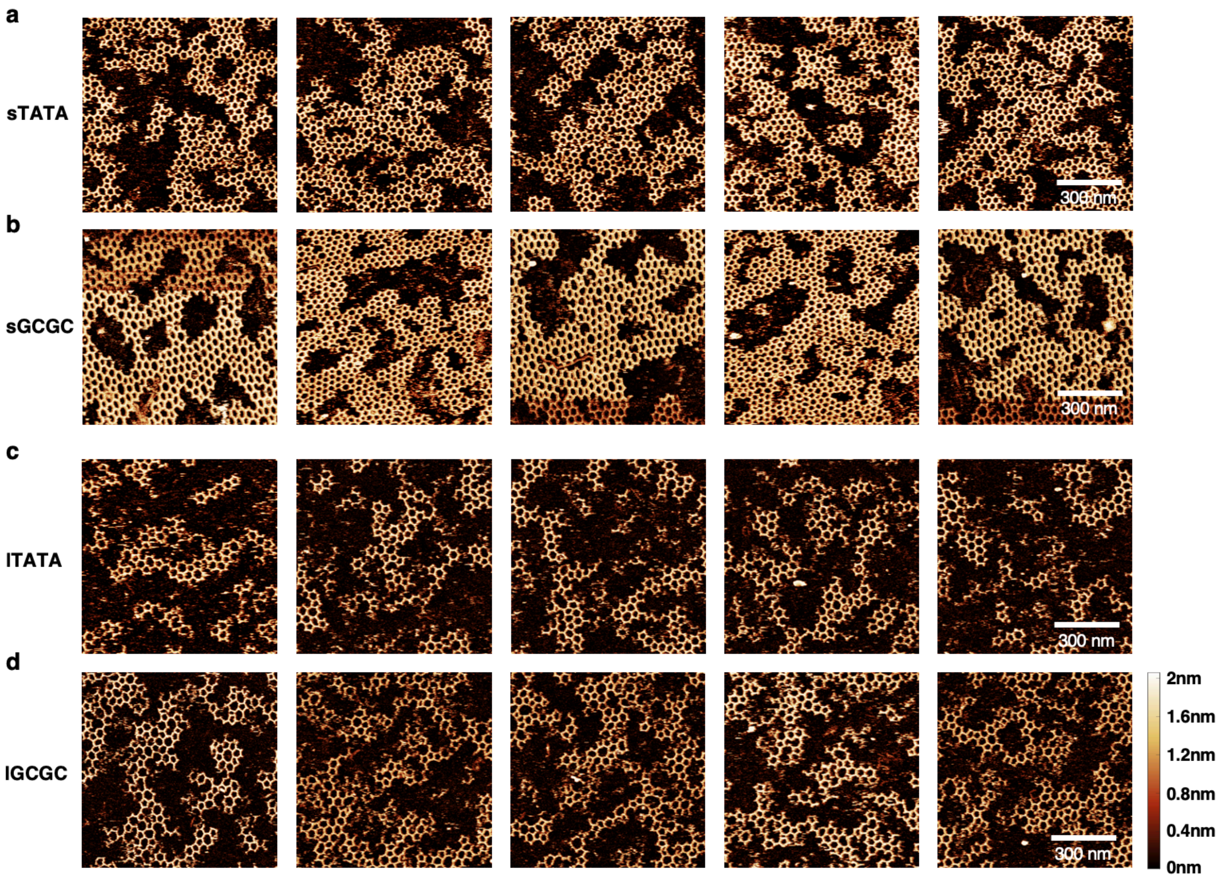

Supplementary Fig. 9. AFM images of sTATA (a), sGCGC (b), lTATA (c), and lGCGC (d). Examples of images used in the anaylsis given in Fig. 2.

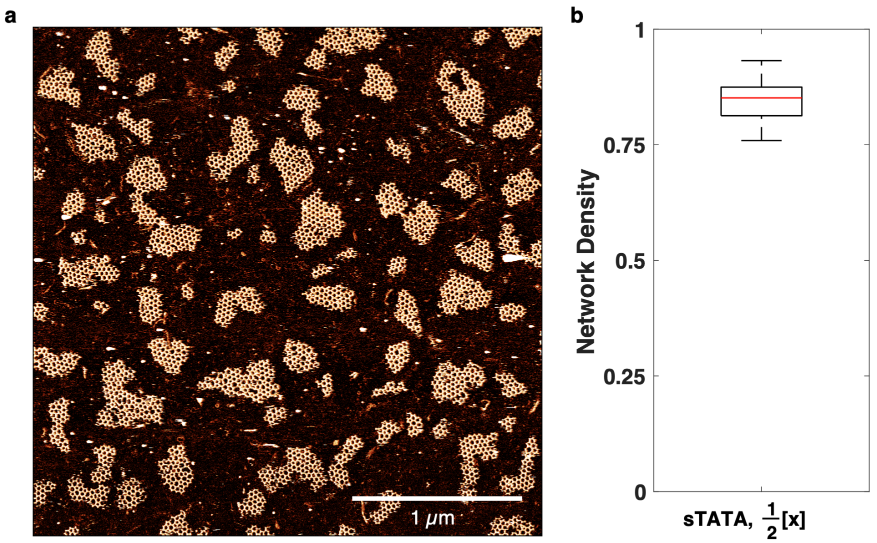

Supplementary Fig. 10. sTATA at half concentration.

While the islands formed by sTATA 3PS exhibit a notably compact structure, the number of islands surpasses that of sGCTA at equivalent concentration (Fig. 1c). Consequently, at higher concentrations, an increased occurrence of nucleation events with sTATA 3PS results in premature coalescence and subsequently leads to a reduction in the overall compactness of the islands that we observe in Fig. 2. The box plot includes the data from 12 regions across the mica surface, each with an area of 750 nm x 750 nm. The box represents the IQR, with the lower and upper edges corresponding to the 25th and 75th percentiles. Whiskers extend to the smallest and largest data points within 1.5 times the IQR from the lower and upper quartiles, respectively. Outliers, defined as data points beyond this range, are shown as individual points. [x] = 6nM

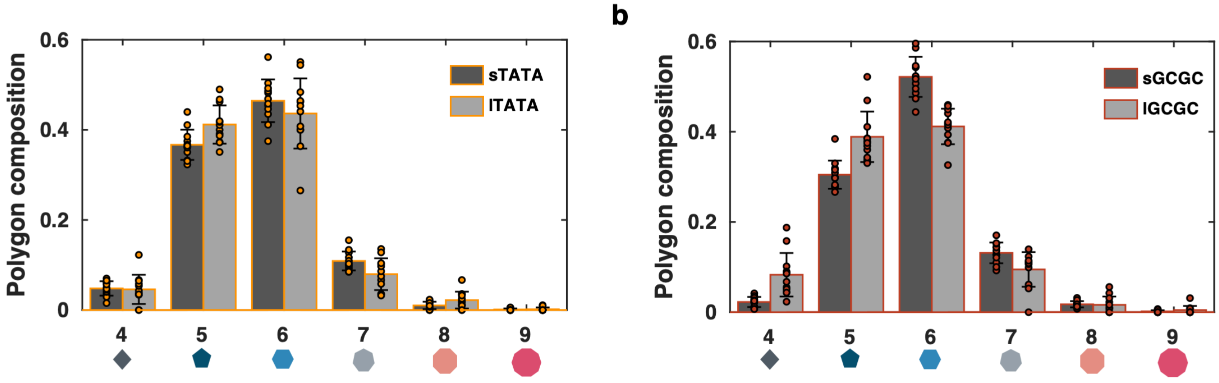

Supplementary Fig. 11. Polygon compositions at the steady state for (a) TATA and (b) GCGC.

The polygon composition of TATA and GCGC 3PS after a day of incubation on mica. The bars include the data of 12 images (750 nm x 750 nm) per condition and they present a weighted mean based on the monomers observed in an image. Error bars represent mean values +/- standard deviation.

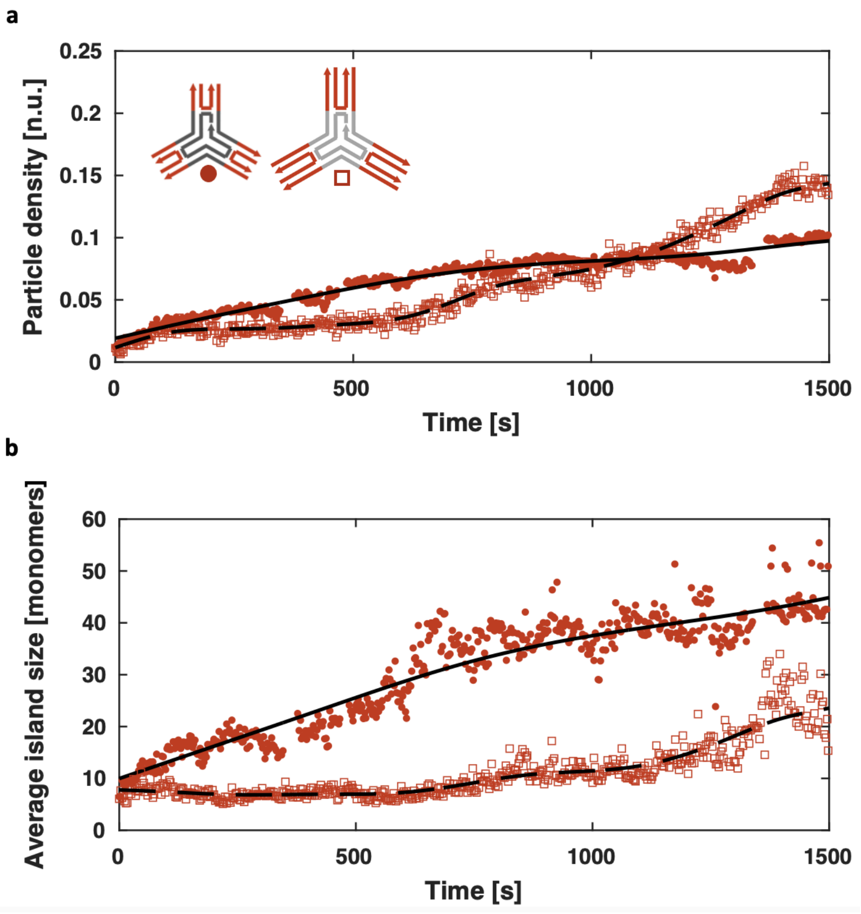

Supplementary Fig. 12. Dynamic self-assembly of short and long GCGC.

a, Particle density on mica as a function of time. The number of particles on surface monotonically increases for both long and short 3PS. **b**, Average island size as a function of time. The islands formed by the short 3PS continuously increases with the arrival of more monomers on the surface. This strongly supports the growth phase of the assembly mechanisms, with every new monomer attaching to an existing island. Contrary, the islands formed by the long 3PS lack any kind of growth, meaning all incoming monomers try to make new nuclei. This changes around 1000s, where the surface density is so high, the small islands start to coalesce.

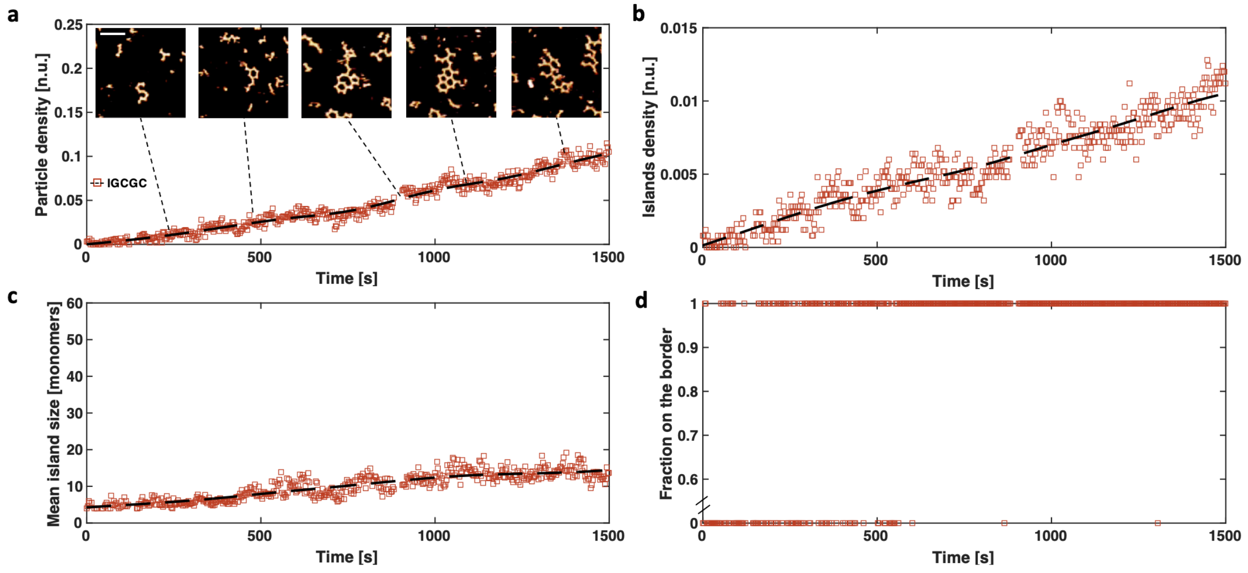

Supplementary Fig. 13. Dynamic self-assembly of lGCGC 3PS. Example 2.

a, Particle density on mica as a function of time and snapshots (~300x300 nm^2) at various stages of the assembly. Scale bar: 100nm **b,** Island density as a function of time. **c**, Mean island size as a function of time. **d,** The fraction of polygons on a border over the total number of polygons in the largest observed island. Black lines are fitted splines.

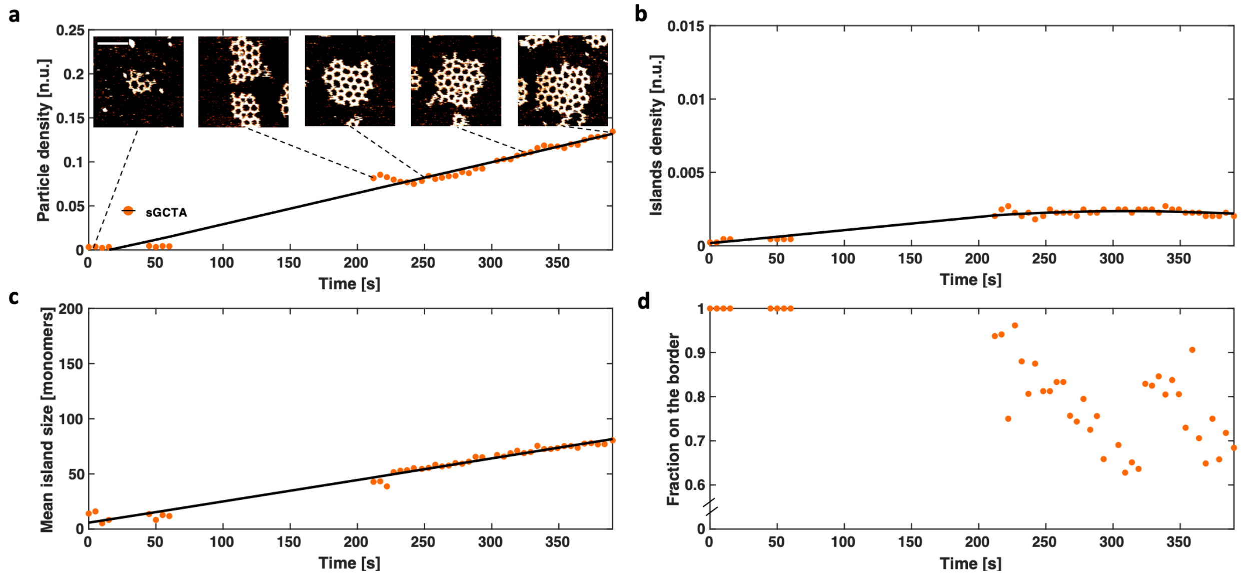

Supplementary Fig. 14. Dynamic self-assembly of sGCTA 3PS. Example 1.

a, Particle density on mica as a function of time and snapshots (~300x300 nm^2) at various stages of the assembly. Scale bar: 100nm **b,** Island density as a function of time. **c**, Mean island size as a function of time. **d,** The fraction of polygons on a border over the total number of polygons in the largest observed island. Black lines are fitted splines.

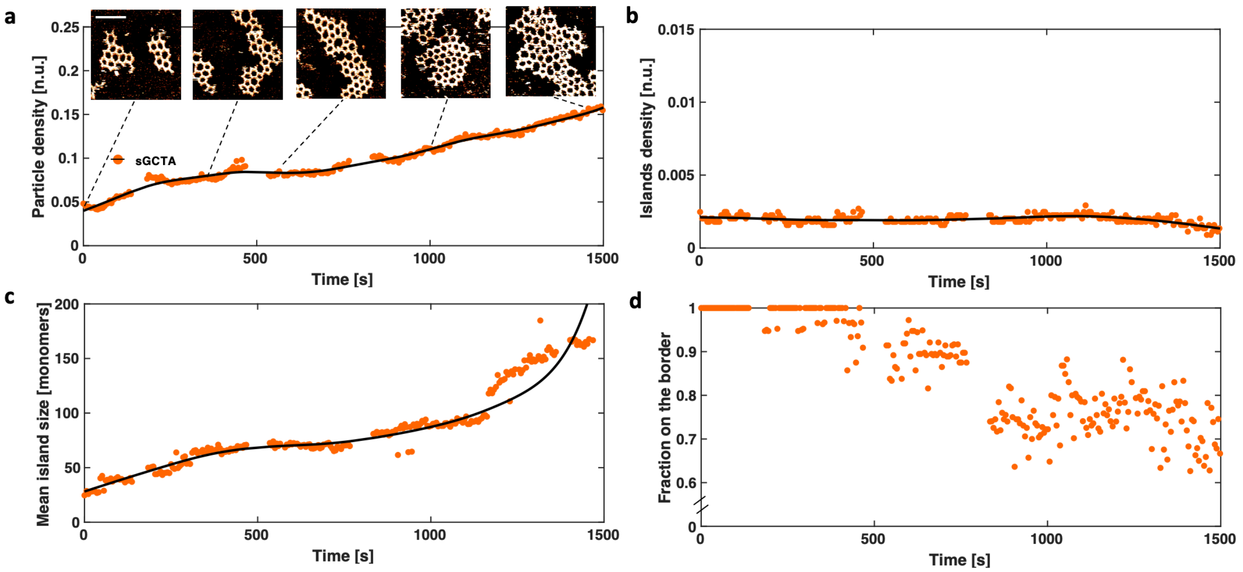

Supplementary Fig. 15. Dynamic self-assembly of sGCTA 3PS. Example 2.

a, Particle density on mica as a function of time and snapshots (~300x300 nm^2) at various stages of the assembly. Scale bar: 100nm **b,** Island density as a function of time. **c**, Mean island size as a function of time. **d,** The fraction of polygons on a border over the total number of polygons in the largest observed island. Black lines are fitted splines.

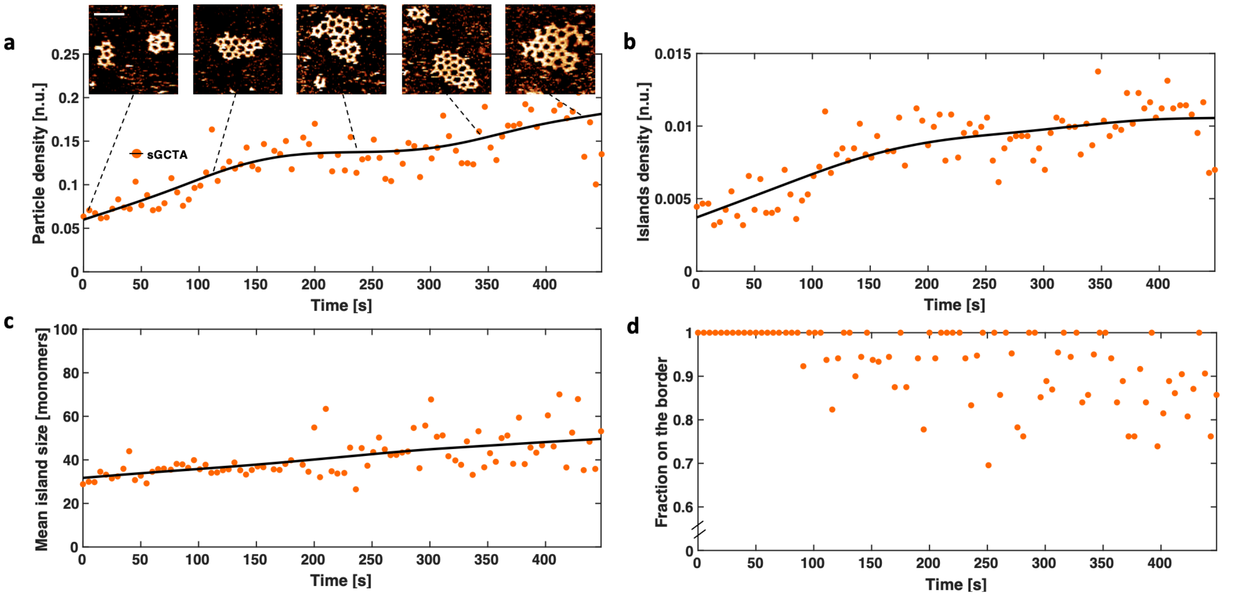

Supplementary Fig. 16. Dynamic self-assembly of sGCTA 3PS. Example 3.

a, Particle density on mica as a function of time and snapshots (~300x300 nm^2) at various stages of the assembly. Scale bar: 100nm **b,** Island density as a function of time. **c**, Mean island size as a function of time. **d,** The fraction of polygons on a border over the total number of polygons in the largest observed island. Black lines are fitted splines.

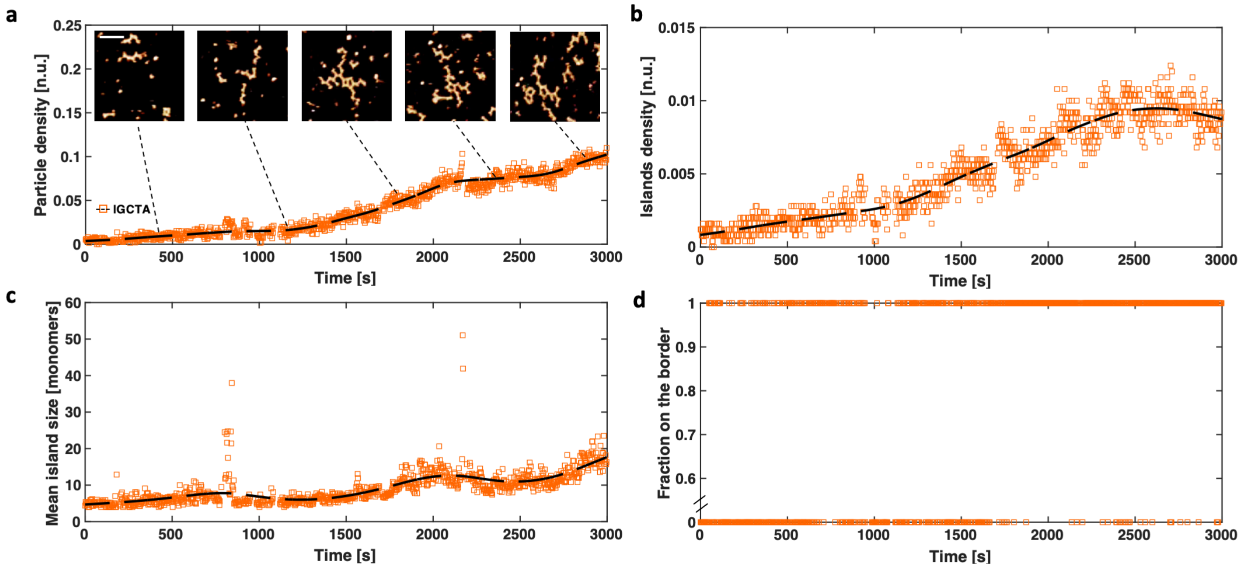

Supplementary Fig. 17. Dynamic self-assembly of lGCTA 3PS. Example 1.

a, Particle density on mica as a function of time and snapshots (~300x300 nm^2) at various stages of the assembly. Scale bar: 100nm **b,** Island density as a function of time. **c**, Mean island size as a function of time. **d,** The fraction of polygons on a border over the total number of polygons in the largest observed island. Black lines are fitted splines.

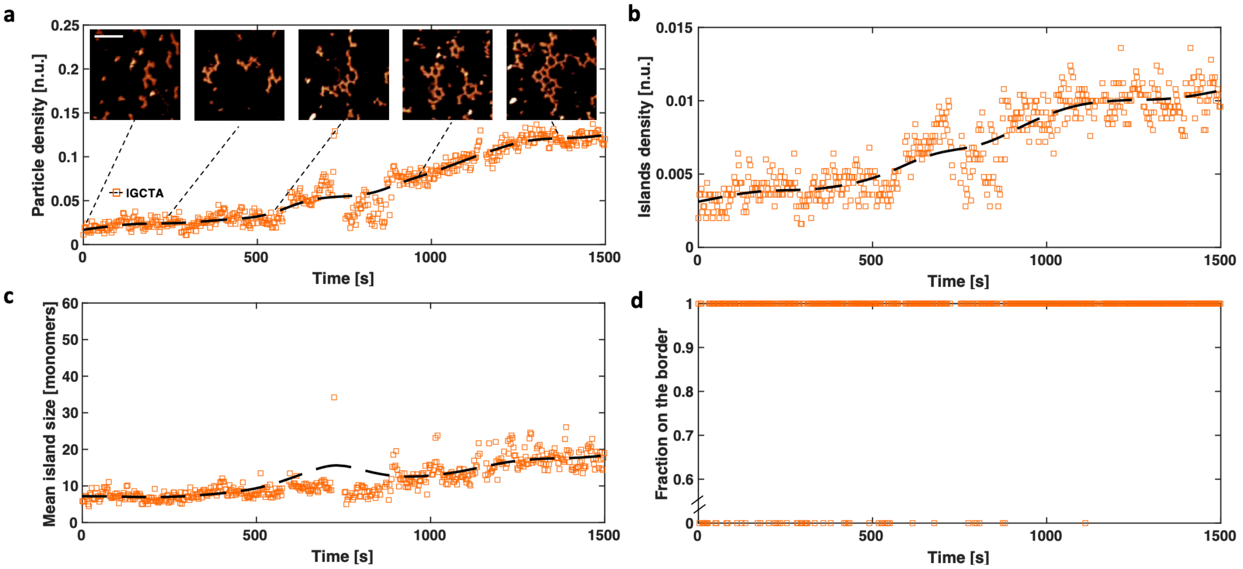

Supplementary Fig. 18. Dynamic self-assembly of lGCTA 3PS. Example 2.

a, Particle density on mica as a function of time and snapshots (~300x300 nm^2) at various stages of the assembly. Scale bar: 100nm **b,** Island density as a function of time. **c**, Mean island size as a function of time. **d,** The fraction of polygons on a border over the total number of polygons in the largest observed island. Black lines are fitted splines.

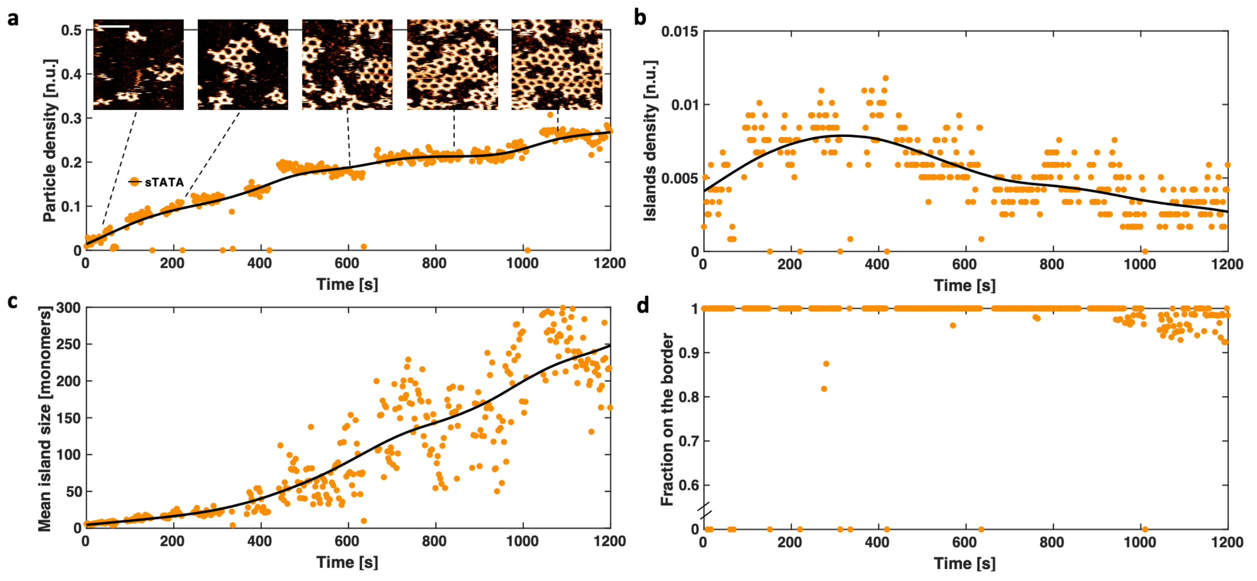

Supplementary Fig. 19. Dynamic self-assembly of sTATA 3PS.

a, Particle density on mica as a function of time and snapshots (~300x300 nm^2) at various stages of the assembly. Scale bar: 100nm **b,** Island density as a function of time. **c**, Mean island size as a function of time. **d,** The fraction of polygons on a border over the total number of polygons in the largest observed island. Black lines are fitted splines.

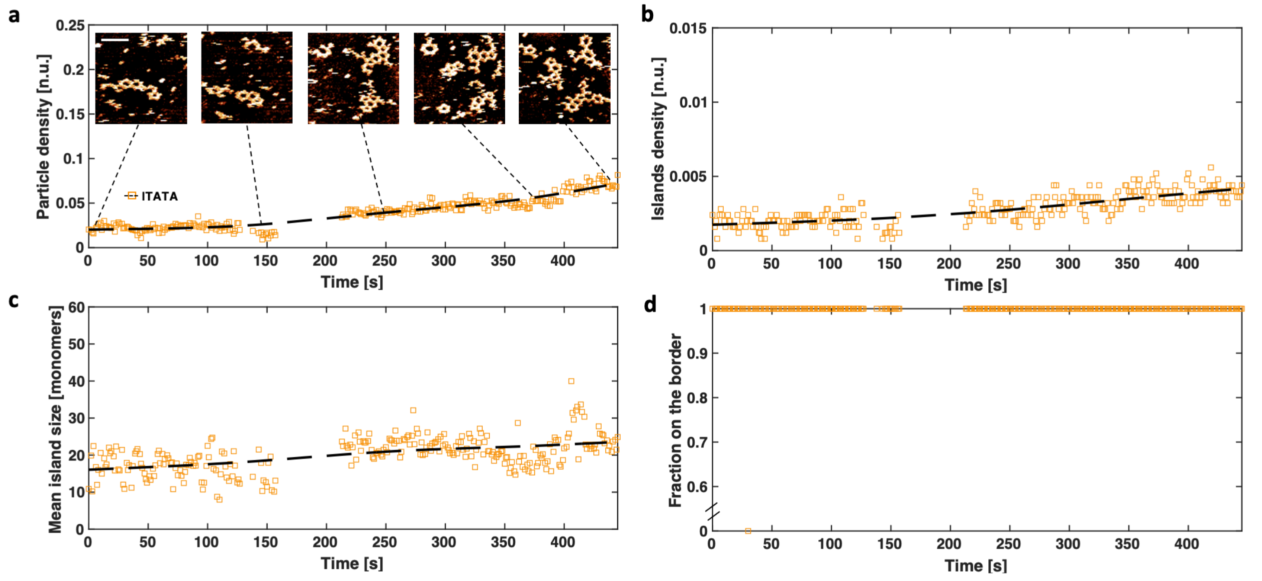

Supplementary Fig. 20. Dynamic self-assembly of lTATA 3PS. Example 1.

a, Particle density on mica as a function of time and snapshots (~300x300 nm^2) at various stages of the assembly. Scale bar: 100nm **b,** Island density as a function of time. **c**, Mean island size as a function of time. **d,** The fraction of polygons on a border over the total number of polygons in the largest observed island. Black lines are fitted splines.

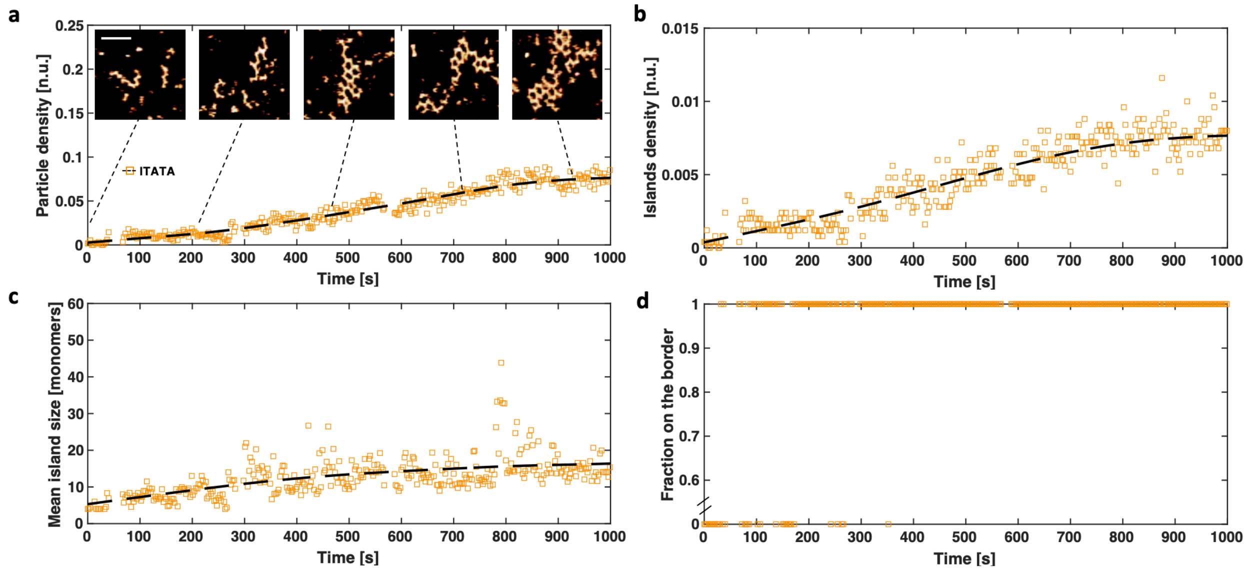

Supplementary Fig. 21. Dynamic self-assembly of ITATA 3PS. Example 2.

a, Particle density on mica as a function of time and snapshots (~300x300 nm^2) at various stages of the assembly. Scale bar: 100nm **b,** Island density as a function of time. **c**, Mean island size as a function of time. **d,** The fraction of polygons on a border over the total number of polygons in the largest observed island. Black lines are fitted splines.

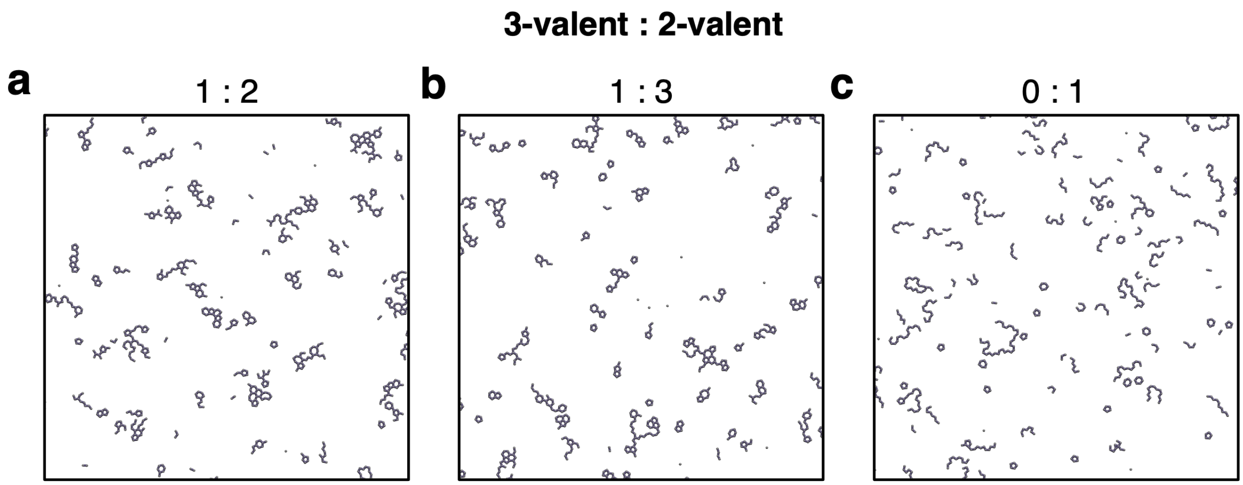

Supplementary Fig. 22. Monte Carlo simulation snapshots of patchy-particles with a 3-valent:2-valent ratio of 1:2 (a), 1:3 (b), and 0:1 (c).

As the ratio of 3-valent to 2-valent particles decreases, the monomers assemble into more linear aggregates rather than radial ones. $\sigma=1$, $\delta=0.038$, $\theta_{pw}=0.3$, and $P_o=0$.

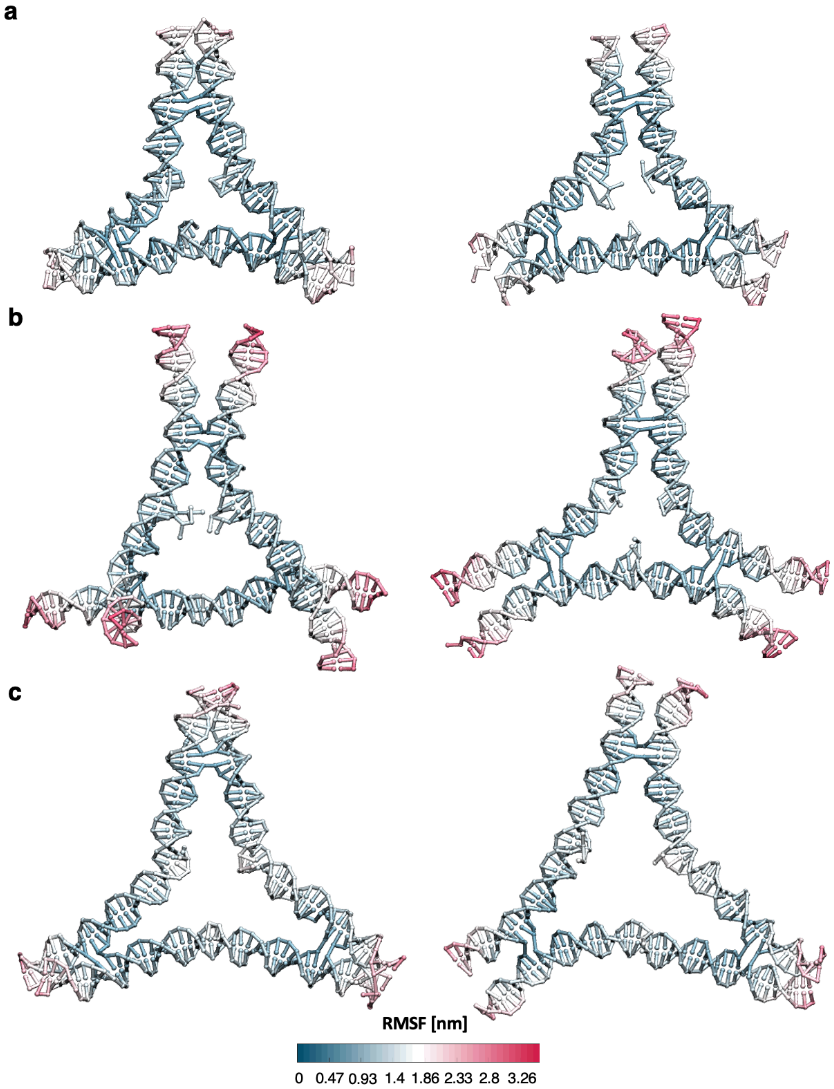

Supplementary Fig. 23. OxDNA simulation frames of (a) short, (b) long and (c) long rigid DNA 3PS in solution.

The left frames display the conformations with the lowest RMSD when compared to the average conformation. The right frames depict the conformations closest to the flat ones.

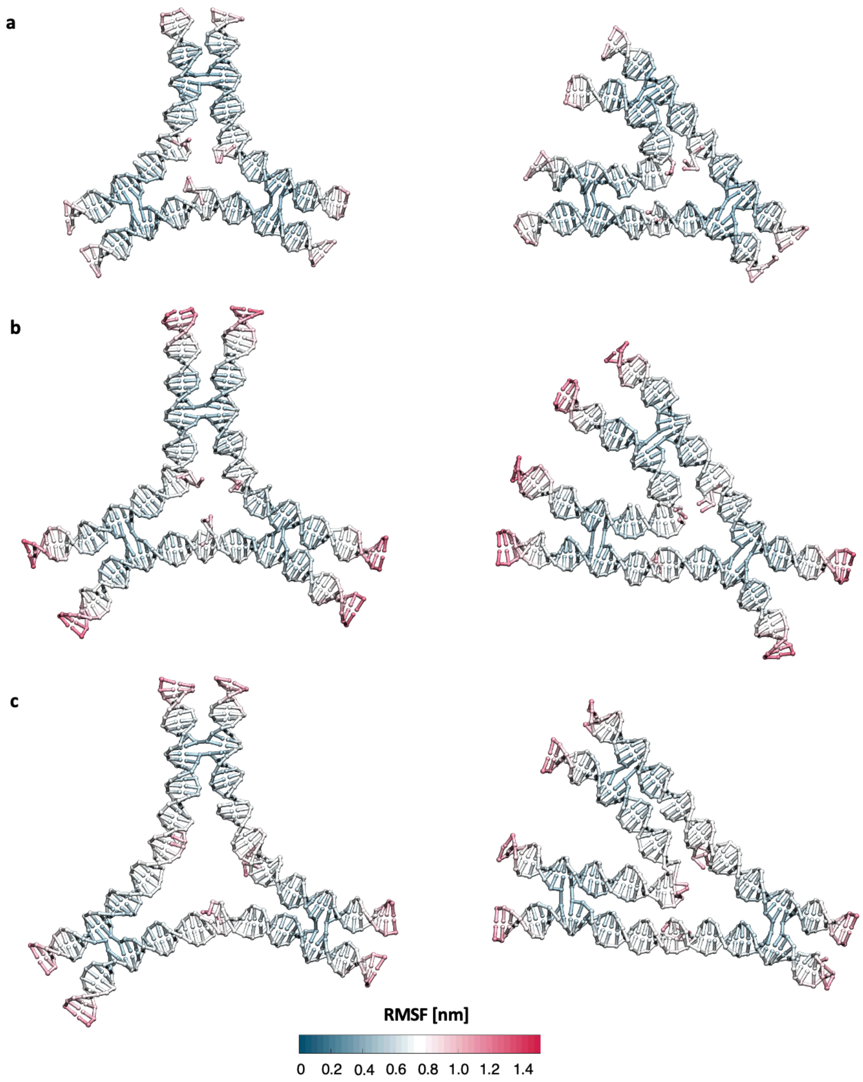

Supplementary Fig. 24. OxDNA simulation frames of (a) short, (b) long and (c) long rigid DNA 3PS confined on the surface.

The left frames display the conformations with the lowest Root Mean Square Deviation (RMSD) when compared to the average conformation. The right frames depict the conformations with the highest RMSD which are less likely to be observed.

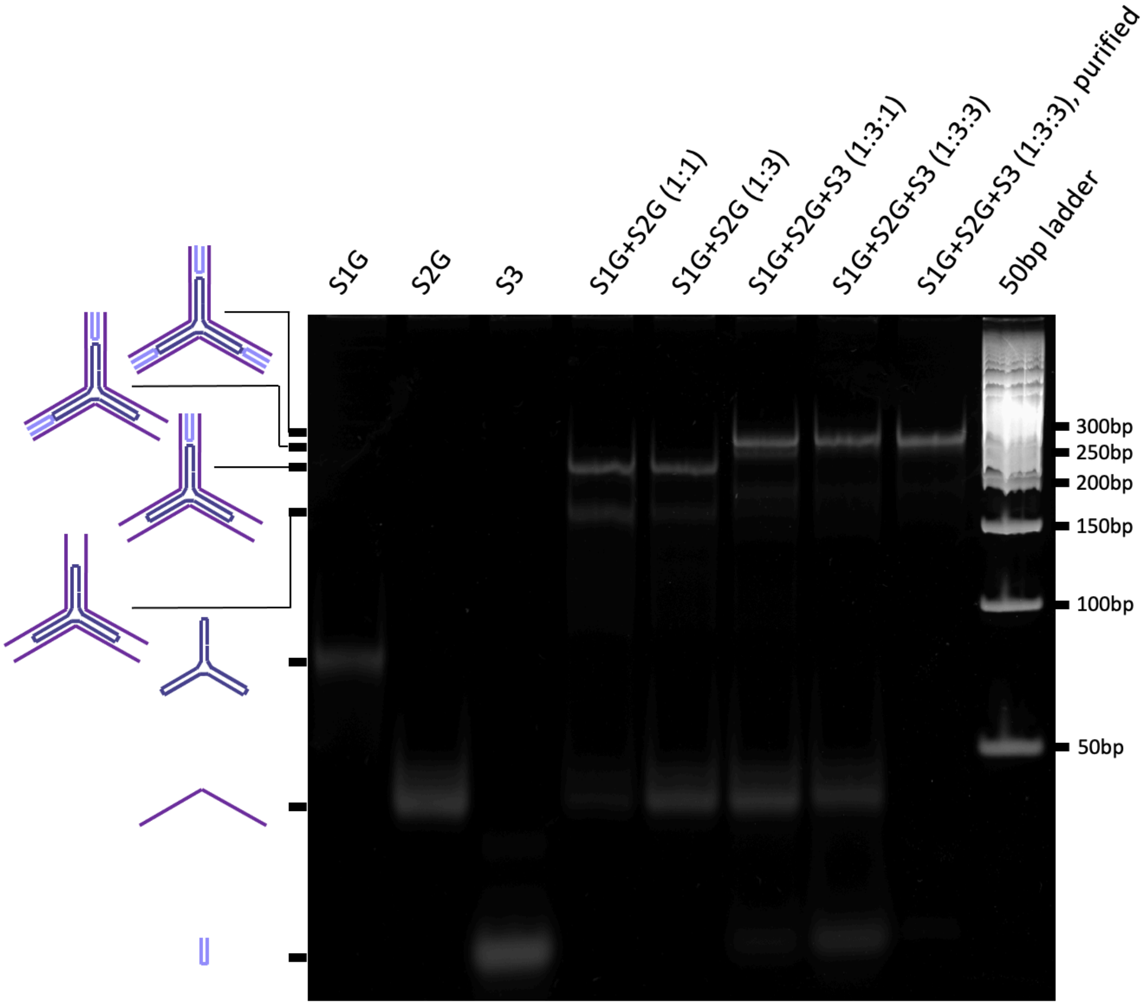

Supplementary Fig. 25. Native PAGE (6%) analysis of the formation of long rigid (LR) 3PS. The compositions of the samples and the structures corresponding to each band are shown above and on left of the gel respectively.

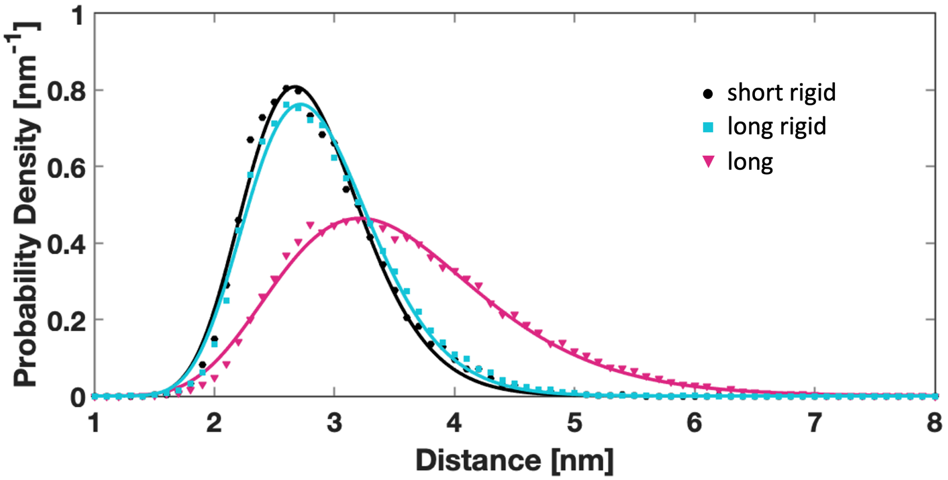

Supplementary Fig. 26. Probability distribution function of 3PS end-to-end distances based on OxDNA simulations.

Each 3PS was forced to be in a plane as explained in the Extended Methods. The data for the short and long are already reported in Fig. 4b. Long rigid 3PS having a similar end-to-end distance distribution to short 3PS is a strong indication that their interface flexibility is similar.

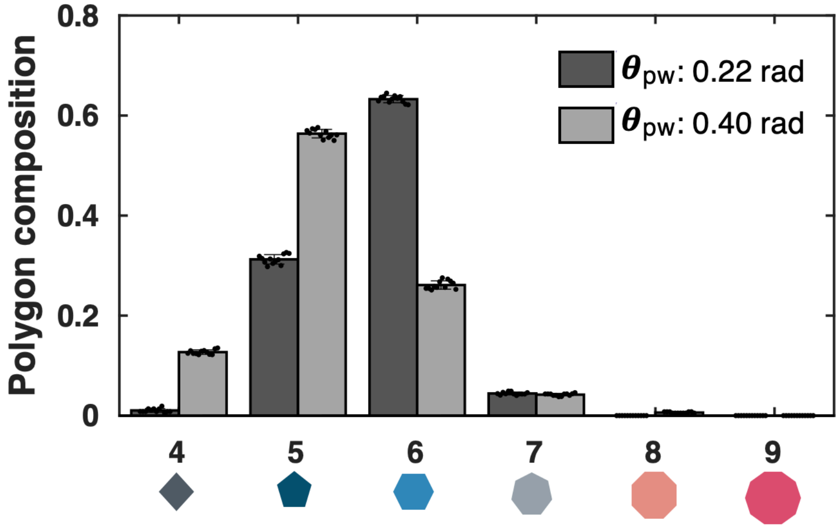

Supplementary Fig. 27. Polygon composition in the patchy particle simulations.

In this set of simulations ε , P_o were 6 and 0.3 respectively. Since the fraction of hexagons we measure *in vitro* is ~0.5 which is included in the interval observed *in silico* (0.24<x<0.67), we expect the patch widths that correctly represent our constructs to be bound between 0.22 and 0.40 radians. Each datapoint represents a simulation frame, corresponding to a 750 nm x 750 nm region from AFM experiments, with a total of 12 frames selected after the convergence of each simulation. Error bars represent mean values +/- standard deviation.

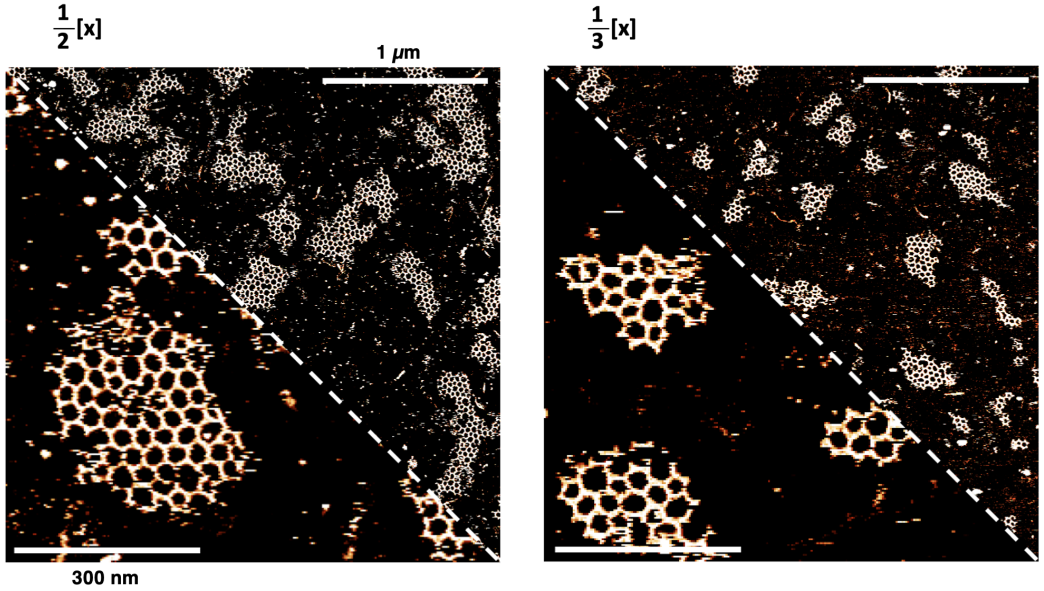

Supplementary Fig. 28. AFM images of the long rigid 3PS self-assembly at lower concentrations.

Long rigid 3PS assembles into radial islands at lower concentrations demonstrating its similar assembly mechanisms as short 3PS. [x] = 3.8nM

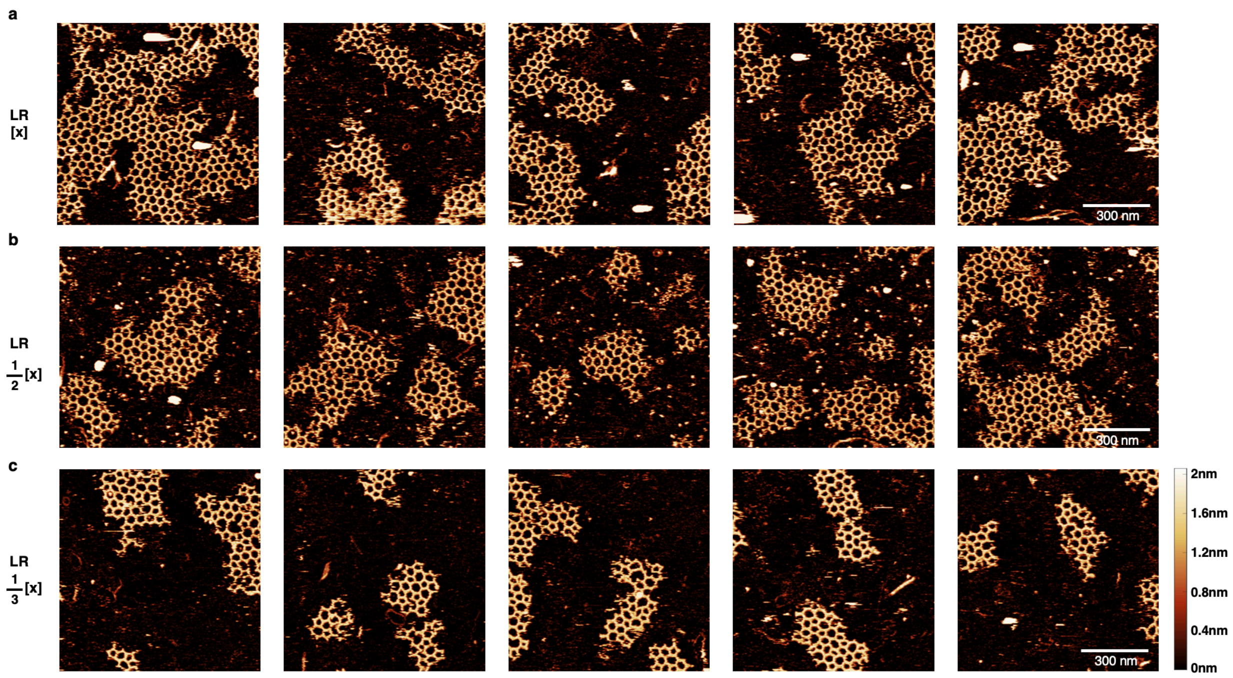

Supplementary Fig. 29. AFM images of long rigid at [x] (a), 1/2[x] (b), and 1/3[x] concentrations.

Examples of images used in the anaylsis given in Fig. 5. [x] = 3.8nM

Supplementary Table 1. DNA motifs and the base sequences of the strands.

Motif	Strand	Sequence
Present in all motifs except long rigid	**1**	5’-AGGCACCATCGTAGGTTTCTTGC<br>CAGGCACCATCGTAGGTTTCTTGCC<br>AGGCACCATCGTAGGTTTCTTGCC-3’
**sTATA** 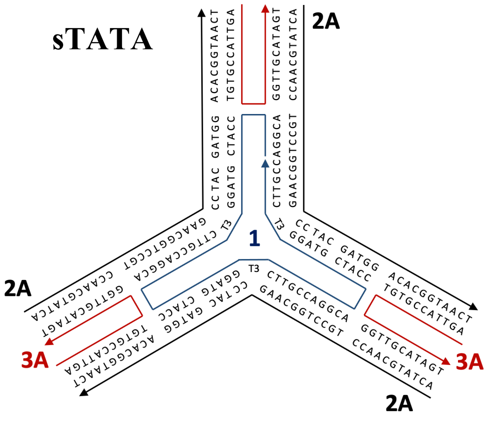	**2A**	5’-ACTATGCAACCTGCCTGGCAAGC<br>CTACGATGGACACGGTAACT-3’
	**3A**	5’-AGTTACCGTGTGGTTGCATAGT-3’
**sGCTA** 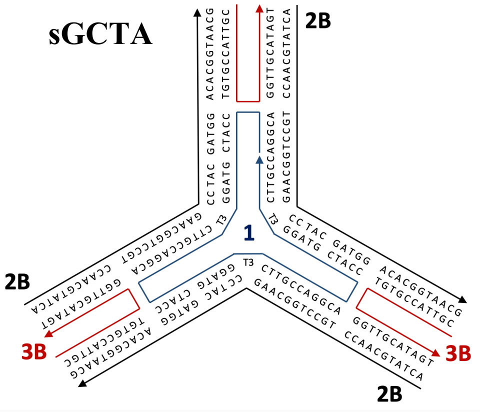	**2B**	5’-ACTATGCAACCTGCCTGGCAAGC<br>CTACGATGGACACGGTAACG-3’
	**3B**	5’-CGTTACCGTGTGGTTGCATAGT-3’
**sGCGC** 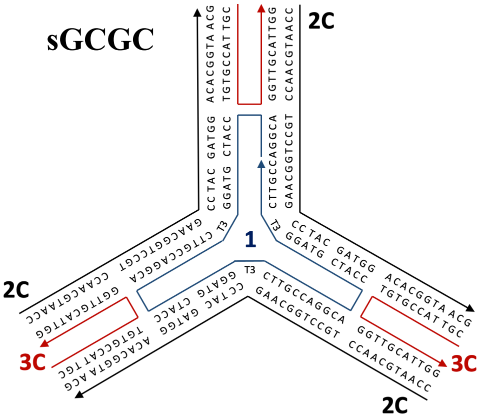	**2C**	5’-CCAATGCAACCTGCCTGGCAAGC<br>CTACGATGGACACGGTAACG-3’
	**3C**	5’-CGTTACCGTGTGGTTGCATTGG-3’

**ITATA** 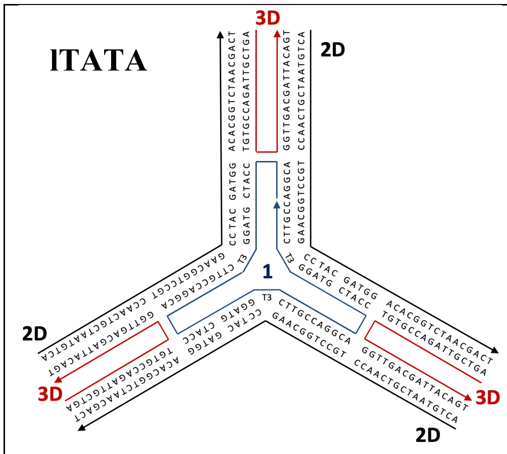	**2D**	5'-ACTGTAATCGTCAACCTGCCTGGC AAGCCTACGATGGACACGGTCTAAC GACT-3'
	**3D**	5'-AGTCGTTAGACCGTGTGGTTGAC GATTACAGT-3'
**IGCTA** 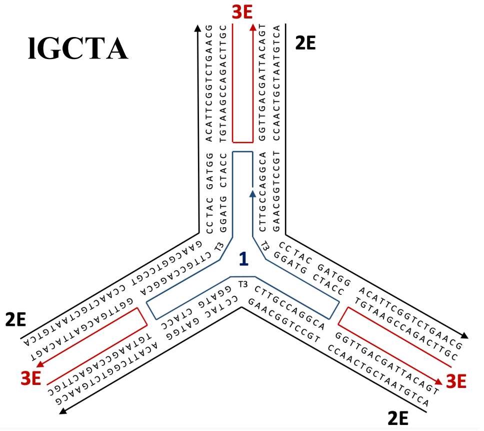	**2E**	5'-ACTGTAATCGTCAACCTGCCTGGC AAGCCTACGATGGACATTCGGTCTG AACG-3'
	**3E**	5'-CGTTCAGACCGAATGTGGTTGA CGATTACAGT-3'
**IGCGC** 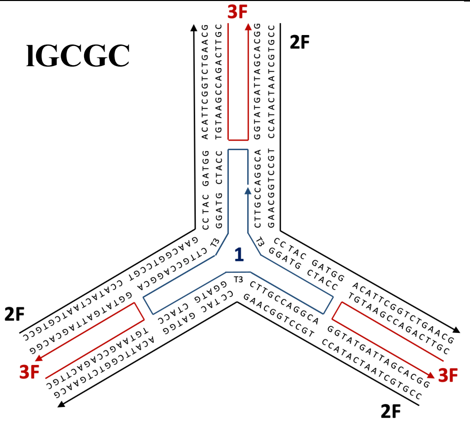	**2F**	5'-CCGTGCTAATCATACCTGCCTGGC AAGCCTACGATGGACATTCGGTCTG AACG-3'
	**3F**	5'-CGTTCAGACCGAATGTGGTATGATT AGCACGG-3'

**Long rigid (LR)** 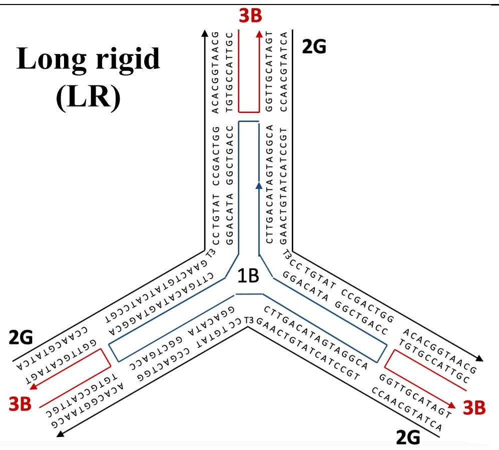	**1B**	5’-AGTAGGCACCAGTCGGATACAGGCTTGACATAGTAGGCACCAGTCGGATACAGGCTTGACATAGTAGGCACCAGTCGGATACAGGCTTGACAT-3’
	**2G**	5’-ACTATGCAACCTGCCTACTATGTCAAGTTTCCTGTATCCGACTGGACACGGTAACG-3’

Supplementary Movies

Movie S1. Dynamic self-assembly of short GCGC

Movie S2. Dynamic self-assembly of long GCGC

Movie S3. Dynamic self-assembly of short GCTA

Movie S4. Dynamic self-assembly of long GCTA

Movie S5. Dynamic self-assembly of short TATA

Movie S6. Dynamic self-assembly of long TATA

Movie S7. Patchy-particle simulation of short

Movie S8. Patchy-particle simulation of long
